# Stress-related hypofrontality in depression and its relation to altered activation prior to the stress response

**DOI:** 10.1016/j.nicl.2026.104019

**Published:** 2026-05-29

**Authors:** Isabell Int-Veen, Ann-Christine Ehlis, Agnes Kroczek, Hendrik Laicher, Andreas J. Fallgatter, David Rosenbaum

**Affiliations:** a Department of Psychiatry and Psychotherapy, Tübingen Center for Mental Health (TüCMH), University of Tübingen, Tübingen, Germany; b German Center for Mental Health, partner site Tübingen, Germany; c LEAD Graduate School & Research Network, University of Tuebingen, Tuebingen, Germany

**Keywords:** Hypofrontality, Prefrontal hypoactivation, Depression, Trier Social Stress Test (TSST), Anticipation

## Abstract

Stress responses unfold across distinct phases, including anticipation, stressor confrontation, and recovery, yet research in depression has predominantly focused on neural processes during stress exposure itself. A robust finding in this context is reduced prefrontal activation under stress in patients with depression (DP). However, neural processes preceding stress exposure may critically shape task-related activation and bias group comparisons. To date, no study has directly examined the impact of this anticipatory neural activation on prefrontal hypoactivation under stress. Here, data from two functional near-infrared spectroscopy (fNIRS) studies employing the Trier Social Stress Test (TSST) in DP and healthy controls (HC) were pooled. Results revealed pronounced group differences prior to task onset, namely increased activation in the DP group compared to HC. With respect to stress-related brain activation, we replicated the well-established finding of reduced stress-related prefrontal activation in DP compared to HC when activation prior to the beginning of each trial was not accounted for. However, when it was accounted for, group differences in stress-related activation were no longer evident. Together, these findings suggest that apparent prefrontal hypoactivation may partly reflect heightened anticipatory recruitment of regulatory brain regions rather than insufficient engagement during task execution, underscoring the importance of (re-)considering the baseline problem in future studies.

## Highlights

• Patients with depression show increased prefrontal activation at baseline.
• Prefrontal hypoactivation is replicated when pre-task baseline is not considered.
• Accounting for baselines eliminates group differences in stress-related activation.

## 1 Introduction

Stress is a common aspect of everyday life. However, individuals differ markedly in how they regulate stress, and these differences have important implications for mental health. In particular, altered stress regulation has been identified as a key factor in the onset, maintenance, and relapse of depressive disorders (Gotlib and Hammen, 2008, Hammen, 2005, Tafet and Nemeroff, 2016). Elucidating the mechanisms that distinguish adaptive from maladaptive stress regulation is therefore critical for advancing our understanding of depression.

Conceptually, stress responses can be divided into three distinct phases: anticipation of a stressor, stressor confrontation, and recovery. To date, much research in depression has focused on aberrant cognitive, affective, and neurophysiological processes during stress exposure itself. For instance, a commonly reported neurobiological finding is reduced prefrontal activation — most prominently in the dorsolateral prefrontal cortex (DLPFC) — in patients with depression during tasks requiring emotional or stress regulation (Akiyama et al., 2018, Fitzgerald et al., 2008, Groenewold et al., 2013, Koenigs and Grafman, 2009, Pizzagalli and Roberts, 2022). The DLPFC constitutes a core node of the Fronto-Parietal Network (FPN), which is concerned with the implementation of cognitive control and goal-directed behavior (Menon and D’Esposito, 2022). In depression, deficient recruitment of prefrontal regions and consequently impaired cognitive control is further associated with hyperactivity in limbic regions such as the amygdala, suggesting an imbalance between top-down regulatory and bottom-up affective processes (Gotlib and Hamilton, 2008, Mayberg, 2002, Ochsner et al., 2004).

While a substantial amount of research focused on stressor confrontation, growing evidence suggests that the anticipatory phase plays a particularly important role. Anticipation does not merely precede the stressor but actively shapes the individual’s response during stress exposure and influences subsequent recovery. This perspective is formalized in the Neurocognitive Framework for Regulation Expectation (NFRE; De Raedt and Hooley, 2016), which proposes that, in healthy individuals, proactive anticipation of a stressor is associated with sustained activation of the DLPFC (Braver, 2012, Herwig et al., 2007, Sohn et al., 2007, Vassena et al., 2019). This activation is thought to exert top-down regulatory control over limbic regions such as the amygdala and to modulate hypothalamic–pituitary–adrenal (HPA) axis responses, ultimately resulting in a more adaptive and attenuated physiological stress response.

In contrast, the NFRE suggests that patients with depression (DP) exhibit altered anticipatory processes, partly due to reduced expectations regarding their ability to cope with future stressors and may result in reduced anticipatory DLPFC activation or, alternatively, increased activation in ventromedial prefrontal regions (VMPFC).

Initial empirical work provides support for this framework. For example, questionnaire-based and network-analytic approaches have yielded first evidence linking anticipatory processes to stress regulation (Pulopulos et al., 2022). Experimental studies using the Trier Social Stress Test (TSST) — a well-established paradigm for inducing acute psychosocial stress involving public speaking and mental arithmetic (Allen et al., 2017) with high ecological validity (Henze et al., 2017) — further suggest that anticipation plays a critical role. Specifically, Pulopulos et al. (2020) reported that individuals who anticipated the TSST as less threatening, based on higher perceived coping ability, exhibited lower cortisol responses and more adaptive autonomic regulation. Similarly, Gaab et al. (2005) demonstrated that a questionnaire assessing subjective anticipatory cognitive appraisal prior to the TSST influences physiological stress responses in terms of lower stress-induced cortisol responses. These findings were further extended by Nasso et al. (2019) who experimentally manipulated this anticipatory cognitive appraisal prior to the TSST: Participants instructed to adopt a reappraisal strategy during preparation for a mock job interview showed higher heart rate variability compared to those instructed to catastrophize, despite, however, no differences in subjective stress.

Crucially, these studies did not assess neural activation, leaving it unclear whether the observed effects of anticipation on physiological stress responses are accompanied by corresponding changes in prefrontal activity. They nevertheless indirectly highlight a critical methodological challenge in the analysis of task-related neural activation: the role of baseline definition. In event-related designs, neural responses are typically quantified relative to a pre-task baseline. However, if this baseline period already contains anticipatory activation, as suggested there, group differences in anticipation may systematically bias estimates of task-related activity.

To illustrate, if anticipatory (DLPFC) activation is reduced in patients with depression, as proposed by the NFRE, task-related hypoactivation may even be underestimated. Conversely, if anticipatory activation would be elevated, subtracting this heightened baseline may artificially attenuate the observed task response, leading to an overestimation of hypoactivation. Thus, differences in anticipatory activity can fundamentally alter the interpretation of task-related neural responses, highlighting a more general problem concerning the choice of baseline and comparison conditions in neuroimaging studies (e.g., Newman et al., 2001, Stark and Squire, 2001). Patterns of brain activation in neuroimaging are strongly dependent on the choice of baseline condition, even when the experimental task remains identical. This finding highlights a key limitation of subtractive logic, showing that observed activations are inherently relative and can vary systematically depending on the cognitive processes involved in the baseline task.

To the knowledge of the authors, no study so far investigated the impact of anticipatory DLPFC activation on the typically observed hypofrontality during the TSST (Henze et al., 2023, Int-Veen et al., 2023, Int-Veen et al., 2026, Int-Veen et al., 2026, Laicher et al., 2022, Rosenbaum et al., 2018a, Rosenbaum et al., 2018b, Rosenbaum et al., 2021, Rosenbaum et al., 2024). To address this gap, the present study aimed to examine whether prefrontal hypoactivation during the TSST reflects diminished stress-related recruitment or whether it may be partly shaped by neural processes occurring prior to task onset. We hypothesized that DP would show reduced DLPFC activation during the TSST relative to HC when using a conventional baseline immediately preceding task onset. We further explored whether DP would show aberrant prefrontal activation prior to the stressor and whether observed group differences during the stress task might vary as a function of the temporal window used for baseline correction.

Lastly, in an exploratory analysis we correlated neural activation prior to task onset with subjective and physiological stress responses, including among others, state rumination which has also been associated with prefrontal hypoactivation under stress (Int-Veen et al., 2023, Int-Veen et al., 2026, Laicher et al., 2022, Rosenbaum et al., 2018b, Rosenbaum et al., 2021, Rosenbaum et al., 2024).

## 2 Methods

### 2.1 Sample

Participants included in this analysis were drawn from two separate studies that employed identical recruitment procedures (for CONSORT diagrams see supplementary material S1).

In both, a total of 65 healthy controls (HC; study 1: 23; study 2: 42) and 77 patients with depression (DP; study 1: 22; study 2: 55) were recruited via circular emails, the University Hospital of Tübingen, and local outpatient psychotherapists.

Study eligibility was assessed using telephone interviews. Exclusion criteria encompassed any medical or psychiatric condition that could influence cerebral metabolism, heart rate variability, or cortisol levels (for a full list, see supplementary material S2). All participants were screened by trained psychologists using the Structured Clinical Interview (SCID; First et al., 2015). HC were excluded in the case of any current or past mental disorder while among DP, primary mental disorders other than ICD-10 diagnoses F32.x, F34.1, F33.x or F43.2 were excluded. Additional exclusion criteria for DP included acute suicidal tendencies, extremely severe depressive symptoms indicated by a Beck Depression Inventory BDI-II score > 50 (German version by Hautzinger et al., 2009), emotional instability as assessed by the treating psychologist, and previous decompensation under social stress.

All study procedures were approved by the ethics committee of the University Hospital and University of Tübingen (study 1: project number 673/2019BO1, study 2: project number 159/2018BO1) and were conducted in accordance with the most recent version of the Declaration of Helsinki.

### 2.2 Procedure

The experimental procedures were nearly identical for both studies. Each study, however, included additional time points. In study 1, an additional stress paradigm — the Socially Evaluated Cold Pressor Test — was conducted, with the order of study sessions counterbalanced across participants. In study 2, the session analyzed here, which involved the TSST, was the first of three TSST sessions for the DP group; between sessions, participants underwent a multi-week training aimed at reducing rumination, whereas the HC group completed only this single session.

**Fig. 1:**
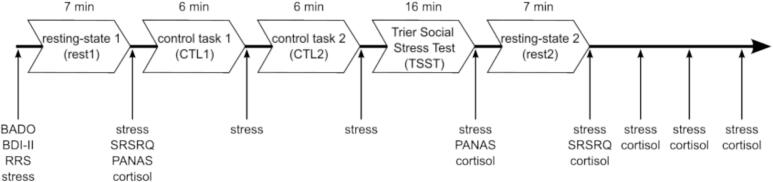
Illustration of the time course of both studies. BADO = questionnaire assessing sociodemographic data, BDI-II = Beck Depression Inventory II, RRS = Ruminative Response Scale, stress = Visual Analogue Scale assessing subjective stress (0–100%), SRSRQ = Stress-Reactive State Rumination Questionnaire, PANAS = Positive and Negative Affect Schedule, cortisol = salivary cortisol sample.

Here, the experimental procedure relevant for the following analysis will be reported (for an overview of the experimental procedure see Fig. 1), while the interested reader is referred to the publications of the corresponding studies for further details (study 1: Rosenbaum et al., 2021, study 2: Rosenbaum et al., 2024). Initially, participants gave written informed consent and completed questionnaires assessing demographic information (BADO), depressive symptoms (Beck Depression Inventory II, BDI-II; German version by Hautzinger et al., 2009), symptoms of social anxiety (Liebowitz Social Anxiety Scale, LSAS; Liebowitz, 1987) and trait rumination (Ruminative Response Scale, RRS; Nolen-Hoeksema and Morrow, 1991). Simultaneously, participants were prepared for the functional near-infrared spectroscopy (fNIRS) and electrocardiogram (ECG) recordings.

Then, after a first assessment of current subjective stress levels using a Visual Analogue Scale (VAS) ranging from 0 to 100%, a 7-minute resting-state (rest1) was conducted. Participants were instructed to sit quietly, allow their mind to wander and keep their eyes open. This was followed by another stress rating, mood assessment (Positive and Negative Affect Schedule PANAS; Crawford and Henry, 2004) and a state rumination questionnaire (Stress-Reactive State Rumination Questionnaire SRSRQ; Int-Veen, n.d.; For items seesupplementary material S3). Further, the first salivary cortisol sample was taken using Salivettes.

Next, two control tasks were performed. In control task 1 (CTL1), participants completed six 40 s trials in which they read aloud sequences of numbers, with 20 s pauses between trials to allow hemodynamic responses to recover. In case participants made an error, they were not interrupted or corrected; instead, errors were documented together with the number of items read.

After CTL1, another stress rating was assessed before control task 2 (CTL2) followed, which also consisted of six 40 s trials and 20 s pauses. During CTL2, participants performed sequential subtractions of 13 from a given starting number at their own pace. Here, any error required the participant to restart the trial. Again, the number of errors and calculations per trial were documented by the study nurse. Although no explicit time pressure was induced during CTL2, the requirement to restart after errors likely introduced a certain degree of social-evaluative threat as well as increased cognitive demands compared to CTL1. In contrast to the TSST panel, however, the study nurse was friendly and non-evaluative, such that participants should be presumably less stressed during the control tasks compared to during the TSST.

After completing the control tasks and another stress rating, two experimenters wearing white lab coats and remaining socially non-responsive entered the room to conduct the stress induction using the Trier Social Stress Test (TSST; Kirschbaum et al., 1993). Participants were asked to imagine they were applying for a position at the University Hospital and were required to deliver a speech about their personal strengths and qualifications. Specifically, they were instructed to choose a role consistent with their own qualifications, which — given the multiprofessional structure of the university hospital and the predominantly student sample — typically resulted in familiar, field-related positions (e.g., student assistant roles). Participants had 5 min time to prepare, after which the experimenters collected their notes and instructed them to stand up and deliver the speech. Following the speech, participants completed a 5-minute arithmetic task similar to CTL2, but were instructed to calculate as quickly and accurately as possible while maintaining eye contact with one experimenter. The second experimenter documented the number of calculations and errors. For both, the mock job interview and the arithmetic task of the TSST, a member of the TSST panel positioned a video camera in front of the participant. Initially, the camera display was oriented toward the participant while the experimenter adjusted the framing to capture the participant’s face, allowing them to see what was being recorded. Subsequently, the display was turned away so that participants could no longer view themselves during the task.

After the TSST, the experimenters turned off the camera, left the room without any comment, and participants completed another stress rating and PANAS, and another cortisol sample was taken.

Then, a second resting-state measurement, analogous to the first, was conducted, before state rumination was assessed. The procedure concluded with a 45-minute post-stress resting period, during which subjective stress and salivary cortisol were assessed every 15 min. For details on recording and preprocessing of salivary cortisol and ECG data we refer to supplementary material S4.

Participants were assessed 60 min after completion of the TSST to ensure their well-being and to verify that stress ratings had returned to baseline levels. If needed, participants were offered the opportunity to talk to a trained psychotherapist. At the end of the study, that is, after completion of all sessions, participants were fully debriefed.

### 2.3 Neural measurements

We used fNIRS as a neuroimaging method as this method is relatively insensitive to motion and speech artifacts (Dieler et al., 2012), which may particularly occur during the TSST. In fNIRS, optodes are placed on the scalp that emit near-infrared light into the brain. Within these wavelength ranges, light can penetrate the skull and reach cortical tissue at a depth of approximately 2–3 cm (Cui et al., 2011, Haeussinger et al., 2011, Haeussinger et al., 2014). In the brain, light is differentially absorbed depending on the concentration of oxygenated and deoxygenated hemoglobin. These changes affect the amount of light reflected back to the detector optodes on the scalp, thereby enabling the measurement of hemodynamic responses. Importantly, fNIRS captures changes in cortical oxygenation that are closely related to the blood-oxygen-level-dependent (BOLD) signal measured with fMRI, reflecting comparable underlying neurovascular processes.

Compared to fMRI, fNIRS offers greater robustness to motion and a more naturalistic testing environment at the cost of lower spatial resolution and limited depth sensitivity, while in contrast to EEG, it provides better spatial localization of cortical activity but lower temporal resolution, reflecting its reliance on hemodynamic rather than electrophysiological signals. Generally, fNIRS demonstrates high reliability (Plichta et al., 2006, Schecklmann et al., 2008) and validity (Huppert et al., 2006, Plichta et al., 2007).

We assessed cortical oxygenation using an ETG-4000 Optical Topography System with a sampling rate of 10 Hz (46-channel continuous wave multichannel fNIRS; Hitachi Medical Co., Japan). With a fixed inter-optode distance of 3 cm, a total of 28 semiconductor lasers and 15 avalanche photodiodes were integrated in two frontal probesets and one parietal probeset covering our five regions of interest (ROI): left and right Inferior Frontal Gyrus (IFG), left and right DLPFC and somatosensory association cortex (SAC).

Scalp-brain correspondence was estimated based on Okamoto et al., 2004, Okamoto and Dan, 2005 as well as Singh et al. (2005). For a visualization of the probeset placement and the assignment of channels to the ROIs, see supplementary material S5.

Near-infrared light was emitted at two wavelengths (695 ± 20 and 830 ± 20 nm) with 2.0 ± 0.4 mW for each wavelength at each optode. The probesets were integrated in EEG caps and placed according to the 10–20 reference points Fpz and Cz. Relative changes in oxygenated (O_2_Hb) and deoxygenated (HHb) hemoglobin were computed using self-written MATLAB 2017 scripts by means of the modified Beer–Lambert Law (Sassaroli and Fantini, 2004).

Preprocessing included the following steps in the described order: the interpolation of single channels with NaN variance, motion artifact correction using Temporal Derivative Distribution Repair (TDDR; Fishburn et al., 2019), Correlation-based signal improvement (CBSI; Cui et al., 2010) and bandpass-filtering to remove low-frequency baseline-drifts (<0.01 Hz) and high-frequency noise (>0.1 Hz). In order to remove artifacts due to data correction, another channel interpolation followed. Accordingly, the manuscript reports the CBSI-corrected O_2_Hb signal. For completeness, results based on HHb are additionally provided, please note, however, that in the previous studies, we also did not observe prefrontal hypoactivation in depressed patients under stress when considering HHb, which is most probably due to reduced power.

Then, because the fNIRS device did not allow for short-distance optodes, we used a global signal reduction with a spatial Gaussian kernel filter (σ = 40) to account for global systemic artifacts (Zhang et al., 2016) and the signal was z-standardized.

Finally, we calculated event-related averages by averaging cortical oxygenation across the six trials for each of the three tasks (CTL1 = control task 1, CTL2 = control task 2, and TSST = the arithmetic task of the TSST), each applying a 5 s baseline correction.

**Fig. 2:**
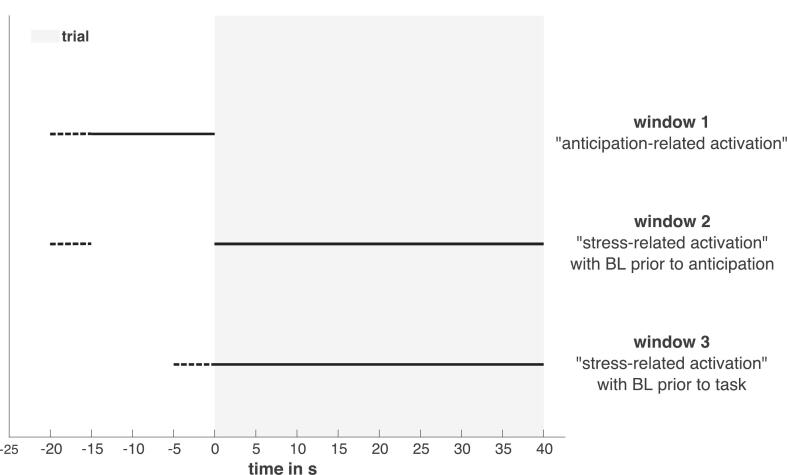
Schematic illustration of the fNIRS time windows extracted (solid line) and baselines (dashed line) used in the present analysis. For each task (CTL1 = control task 1, reading numbers aloud; CTL2 = control task 2, mental arithmetic without TSST panel; TSST = arithmetic task of the TSST, mental arithmetic with TSST panel), the six trials were averaged. Each trial consisted of a 40 s task period (light grey box) followed by a 20 s rest period, resulting in a total task duration of 6 min. Three time windows were extracted. Window 1 captured “anticipatory” activity and spanned from 15 s before task onset (i.e., before reading/calculating) to the actual task onset (0 s). Baseline correction was performed using the 5 s preceding this window (−20 to −15 s; dashed line). Window 2 was used to investigate stress-related hemodynamic responses while controlling for differences in “anticipatory” activity. It covered the task period from 0 to 40 s and used the same baseline as window 1 (−20 to −15 s; dashed line). Window 3 followed the standard approach used in previous studies: task-related hemodynamic responses were extracted from 0 to 40 s, with baseline correction applied using the 5 s immediately preceding task onset (−5 to 0 s; dashed line).

Event-related averages were computed for three different time windows: First, an “anticipatory” time window spanning the 15 s prior to trial onset (−15 to 0 s) using the preceding 5 s as baseline (−20 to −15 s) (window 1 in Fig. 2); second, a time window covering the trial period (0 to 40 s), baseline-corrected using the same baseline as in window 1 (−20 to −15 s) (window 2 in Fig. 2); and third, a time window covering the trial period (0 to 40 s) but using a standard baseline correction based on the 5 s immediately preceding trial onset (−5 to 0 s) (window 3 in Fig. 2). Please note that after the end of each 20 s pause, the next trial did not start immediately. Instead, the start was response-locked and initiated via a key press by the study nurse who was also present, once the next starting number for the upcoming run had been announced — by the study nurse during the control tasks, or by the experimenters during the arithmetic task of the TSST. Due to potential additional instructions (e.g., “Calculate faster” in the TSST), the time point −20 s relative to the start of a run does not always correspond exactly to 60 s after run onset (see Fig. 2 for illustration). Therefore, we decided to define the onset of the anticipation phase at −15 s, which allowed us, in line with the other baseline corrections, to use the preceding 5 s as the baseline period. Finally, data was exported for each time window and ROI.

### 2.4 Data analysis

Data analyses were conducted using IBM SPSS Statistics (version 31). Figures were generated using RStudio (version 2026.01.0 + 392) and R (version 4.5.2), employing the ggplot2 package (Wickham, 2016), as well as MATLAB (version R2025a).

We have made the SPSS syntax and R code for the plots, as well as the basic script for the fNIRS data analysis, publicly available on GitHub (https://github.com/isabellintveen/TSSTAnticipation.git). Please note that the fNIRS analysis relies on numerous MATLAB sub-scripts, which we are unable to release in full.

First, a manipulation check was performed to examine whether the TSST resulted in a significant subjective and physiological stress response. To this end, separate repeated-measures ANOVAs (rmANOVAs) were conducted for subjective stress, negative affect, positive affect, state rumination as well as salivary cortisol, including the within-subjects factor condition (representing the repeated time points of assessment) and the between-subjects factor group (patients with depression; DP vs. healthy controls; HC), as well as their interaction.

Next, neural data were analyzed. For this purpose, identical rmANOVAs were conducted separately for each exported time window (see Fig. 2). These models included the within-subjects factors condition (control task 1; CTL1 vs. control task 2; CTL2 vs. the arithmetic task of the TSST; TSST) and region of interest (ROI: left and right DLPFC, left and right IFG, and SAC), as well as their interactions with the between-subjects factor group (DP vs. HC).

For all rmANOVAs, violations of the sphericity assumption were addressed by applying Greenhouse-Geisser corrections when ε < 0.75 and Huynh-Feldt corrections when ε ≥ 0.75. Before performing the rmANOVAs, multivariate outliers were identified using Mahalanobis distance and excluded if the squared distance exceeded the critical χ^2^ value for *p* < 0.001. No outliers were observed for O_2_Hb. For HHb data, two outliers were detected in the fNIRS stress-related activation measurements, both with the standard and anticipation-corrected baselines. Additionally, three multivariate outliers were excluded for HHb data in the “anticipation” window.

Given that the primary focus was on the hypothesized condition by group interactions, post hoc pairwise comparisons were performed only when this interaction was statistically significant, using Benjamini-Hochberg correction for multiple comparisons. We report which pairwise comparisons remained significant after correction (*p_corr_*), alongside the corresponding uncorrected results (*p*-values and effects sizes). Post hoc tests were not performed for the factor ROI due to different path length factors.

Finally, as an exploratory analysis, correlations were examined between neural activation (O_2_Hb) during window 1 and subjective stress responses (subjective stress, state rumination, negative affect) as well as physiological variables (heart rate and cortisol levels).

Specifically, the following time points were correlated: fNIRS activation during the arithmetic task, as this directly corresponds to the main analysis; subjective stress ratings and negative affect assessed immediately after the TSST, in order to capture responses as close in time to the stressor as possible; and heart rate during the arithmetic task, to provide a physiological measure that is temporally aligned with the fNIRS data. In addition, salivary cortisol levels measured 15 min after the TSST were included, as this time point reflects the peak cortisol response to the stress induction in both groups. Finally, state rumination assessed after the second resting-state measurement was considered, as it represents an index of stress-reactive rumination.

For correlations between neural activation for time windows 2 and 3 with subjective stress responses (subjective stress, state rumination, negative mood) as well as physiological variables (heart rate and cortisol levels) see supplementary material S6.

## 3 Results

### 3.1 Demographic data

Before merging the two datasets, we investigated differences between HC and DP within each study. There were no differences in age in study 1, but we found DP to be on average five years older in study 2 (*M* = 32.60, *SD* = 11.12) compared to HC in study 2 (*M* = 27.29, *SD* = 9.39), *t*(93.99) = 2.548, *p* < 0.01, *d* = 0.510. To ensure the robustness of our results, we conducted the fNIRS analysis with age included as a covariate. The analysis without age as a covariate can be found in supplementary material S7. Note that the results are largely consistent.

In both studies, the proportion of females was comparable among DP and HC. Lastly, in both studies, DP showed elevated trait rumination, as indicated by RRS scores, and elevated symptoms of depression as indicated by higher BDI-II scores (see Table 1).

**Table 1:** Demographic data dependent on the subsamples and the merged total sample.

study	variable	group	*M*	*SD*	test-statistic comparing DP and HC	*p*-value	Cohen's *d*
1	age	DP (*n* = 22)	27.14	6.15	*t*(43) = 1.008	0.319	0.301
		HC (*n* = 23)	25.35	5.75			
	BDI-II	DP (*n* = 22)	24.14	11.85	*t*(22.10) = 8.596	< 0.001	2.619
		HC (*n* = 23)	2.13	1.96			
	RRS	DP (*n* = 22)	2.59	0.50	*t*(43) = 6.447	< 0.001	1.923
		HC (*n* = 23)	1.73	0.39			
	LSAS	DP (*n* = 22)	63.35	26.36	*t*(34.01) = 5.963	< 0.001	1.798
		HC (*n* = 23)	24.54	15.75			
	% female	DP (*n* = 22)	77.27%		$\chi^{2}\left(1\right)$ = 0.006	0.936	
		HC (*n* = 23)	78.26%				
2	age	DP (*n* = 55)	32.60	11.12	*t*(93.99) = 2.548	< 0.01	0.510
		HC (*n* = 42)	27.29	9.39			
	BDI-II	DP (*n* = 55)	26.62	7.29	*t*(91.92) = 18.727	< 0.001	3.619
		HC (*n* = 42)	3.95	4.58			
	RRS	DP (*n* = 55)	2.78	0.44	*t*(94) = 14.415	< 0.001	2.966
		HC (*n* = 42)	1.57	0.37			
	LSAS	DP (*n* = 54)	56.80	23.37	*t*(82.93) = 9.529	< 0.001	1.821
		HC (*n* = 41)	21.62	12.00			
	% female	DP (*n* = 55)	69.09%		$\chi^{2}\left(1\right)$ = 1.091	0.296	
		HC (*n* = 42)	78.57%				
total	age	DP (*n* = 77)	31.04	10.22	*t*(139.80) = 2.856	< 0.01	0.473
		HC (*n* = 65)	26.60	8.29			
	BDI-II	DP (*n* = 77)	25.91	8.82	*t*(108.95) = 20.218	< 0.001	3.217
		HC (*n* = 65)	3.31	3.94			
	RRS	DP (*n* = 77)	2.72	0.46	*t*(139) = 15.270	< 0.001	2.580
		HC (*n* = 65)	1.63	0.38			
	LSAS	DP (*n* = 76)	58.69	24.28	*t*(120.40) = 11.081	< 0.001	1.795
		HC (*n* = 64)	22.67	13.42			
	% female	DP (*n* = 77)	71.43%		$\chi^{2}\left(1\right)$ = 0.921	0.334	
		HC (*n* = 65)	78.47%				

**Note.** study 1 = (Rosenbaum et al., 2021); study 2 = (Laicher et al., 2023, Rosenbaum et al., 2024), BDI-II = Beck Depression Inventory II (Hautzinger et al., 2009); RRS = Ruminative Response Scale (Nolen-Hoeksema and Morrow, 1991), LSAS = Liebowitz Social Anxiety Scale (Liebowitz, 1987), DP = patients with depression, HC = healthy controls.

Overall, most DP (70.1%) were diagnosed with an F32.X diagnosis (depressive episode), 27.3% with an F33.X diagnosis (recurrent depressive episode) and 2.6% with an F43.2 diagnosis (adjustment disorder with depressed mood). For a cross table of ICD-10 diagnoses in each study, see supplementary material S8.

### 3.2 Manipulation check

As in both previous studies, the TSST showed a reliable stress induction in terms of significant increases in subjective stress, negative affect and state rumination due to the TSST and more pronounced increases in DP compared to HC. We further observed significant increases in salivary cortisol, however no group-dependent differences (for results of the rmANOVAs and plots see supplementary material S9).

### 3.3 Stress-related brain activation

First, to investigate stress-related brain activation, we fitted a rmANOVA to the fNIRS data during the tasks using a standard baseline correction just before the trial began (window 3 in Fig. 2) using the within-subjects factors condition (CTL1 vs. CTL2 vs. arithmetic task of the TSST) and ROI (left IFG, right IFG, left DLPFC, right DLPFC, SAC) in interaction with the between-subjects factor group (HC vs. DP) and age as a covariate.

As a result, we observed a significant interaction of condition and group for O_2_Hb (*F*(2, 278) = 4.872, *p* < 0.01, $\eta_{p}^{2}$ = 0.034, HHb: *F*(2, 274) = 1.061, *p* = 0.348, $\eta_{p}^{2}$ = 0.008).

**Fig. 3:**
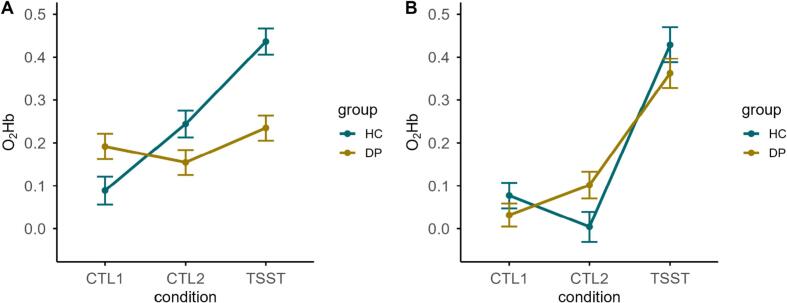
Line plot of the raw data of task-related fNIRS data dependent on condition (CTL1 = control task 1, i.e. reading numbers; CTL2 = control task 2, i.e. mental arithmetics without TSST panel; TSST = arithmetic task of the TSST, i.e. mental arithmetics with TSST panel) for two groups: HC (healthy controls) and DP (patients with depression) using the standard baseline correction just before the trial began (window 3 in figure 2) (A) and the baseline correction prior to the beginning of the trial (window 2 in figure 2) (B). Lines depict the mean cui-corrected O_2_Hb signals for each group, error bars represent +/- 1 standard error of the mean (SE).

**Fig. 4:**
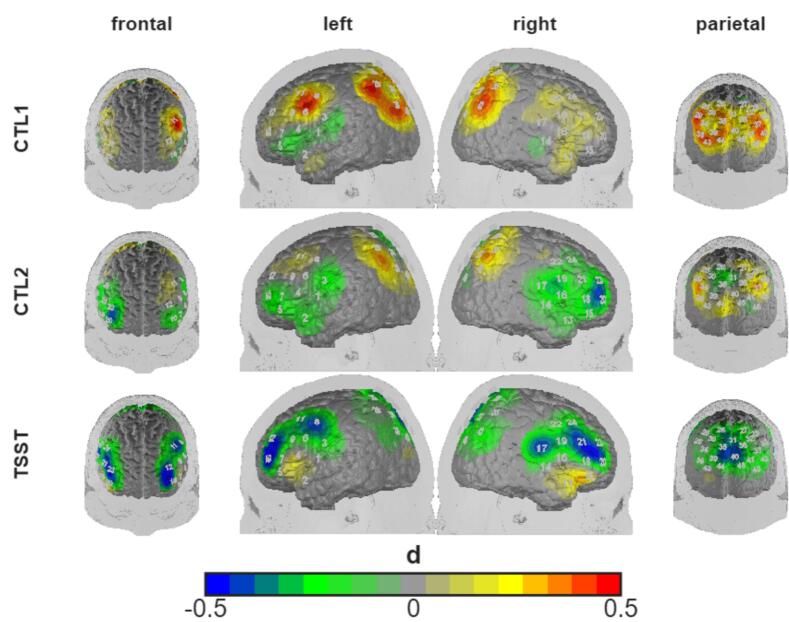
Differences in cortical oxygenation (O_2_Hb) between HC (healthy controls) and DP (patients with depression) dependent on condition (CTL1 = control task 1, i.e. reading numbers; CTL2 = control task 2, i.e. mental arithmetics without TSST panel; TSST = arithmetic task of the TSST, i.e. mental arithmetics with TSST panel) using the standard baseline correction just before the trial began (window 3 in figure 2). Cool colors indicate reduced O_2_Hb-levels in DP as compared to HC; warm colors vice versa. Differences are depicted in Cohen's *d*. Numbers indicate fNIRS channels. left IFG = channel 6, 9, 7; right IFG = channel 18, 19, 21; left DLPFC = channel 10, 11, 12; right DLPFC = 20, 23, 24; SAC = channel 25, 26, 27, 28, 30, 31, 32, 35, 36.

Benjamini-Hochberg corrected pairwise comparisons of the interaction of condition and group indicated no significant (*p_corr_* < 0.05) differences between the groups at any time point but when considering differences between consecutive conditions, we observed significant increases in O_2_Hb between CTL1 and CTL2 (mean difference = -0.161, CI [-0.294;–0.027], *SE* = 0.068, *p* < 0.05) and between CTL2 and the arithmetic task of the TSST but only in HC (mean difference = -0.174, CI [-0.300;–0.049], *SE* = 0.063, *p* < 0.01) (see Fig. 3A). For brainmaps see Fig. 4.

Lastly, we observed a significant lower-order main effect of condition for O_2_Hb (*F*(2, 278) = 3.797, *p* < 0.05, $\eta_{p}^{2}$ = 0.027, HHb: *F*(2, 274) = 0.388, *p* = 0.679, $\eta_{p}^{2}$ = 0.003).

No significant interaction with or main effect of age occurred for O_2_Hb. For HHb, we observed a significant main effect of age, *F*(1, 137) = 1.078, *p* < 0.01, $\eta_{p}^{2}$ = 0.069.

Then, we fitted the same rmANOVA on fNIRS data of window 2. Here, we did not observe a significant interaction of condition and group (O_2_Hb*: F*(2, 278) = 1.046, *p* = 0.353, $\eta_{p}^{2}$ = 0.007, HHb: *F*(2, 274) = 1.230, *p* = 0.294, $\eta_{p}^{2}$ = 0.009) (see Fig. 3B).

There was a significant interaction of condition and ROI (O_2_Hb*: F*(6.521, 906.387) = 2.349, *p* < 0.05, $\eta_{p}^{2}$ = 0.017, HHb: *F*(6.113, 837.545) = 3.054, *p* < 0.01, $\eta_{p}^{2}$ = 0.022), as well as significant lower-order main effects of condition (O_2_Hb*: F*(2, 278) = 6.908, *p* < 0.01, $\eta_{p}^{2}$ = 0.047, HHb: *F*(2, 274) = 4.915, *p* < 0.01, $\eta_{p}^{2}$ = 0.035) and ROI but only for O_2_Hb (*F*(3.481, 483.798) = 12.128, *p* < 0.001, $\eta_{p}^{2}$ = 0.080, HHb: *F*(3.666, 461.099) = 1.128, *p* = 0.340, $\eta_{p}^{2}$ = 0.008).

No significant interaction with or main effect of age occurred.

For a visualization of the time series of window 2 and 3 see supplementary material S10.

### 3.4 Anticipation-related brain activation

Next, we fitted a rmANOVA to investigate the neural activation in both groups prior to the onset of the trial (window 1) using the within-subjects factor condition (CTL1 vs. CTL2 vs. arithmetic task of the TSST), ROI (left IFG, right IFG, left DLPFC, right DLPFC, SAC) in interaction with the between-subjects factor group (HC vs. DP) and age a covariate.

As a result, we observed a significant interaction of condition and group for O_2_Hb, *F*(2, 278) = 4.129, *p* < 0.05, $\eta_{p}^{2}$ = 0.029 (HHb: *F*(2, 272) = 0.979, *p* = 0.377, $\eta_{p}^{2}$ = 0.007).

**Fig. 5:**
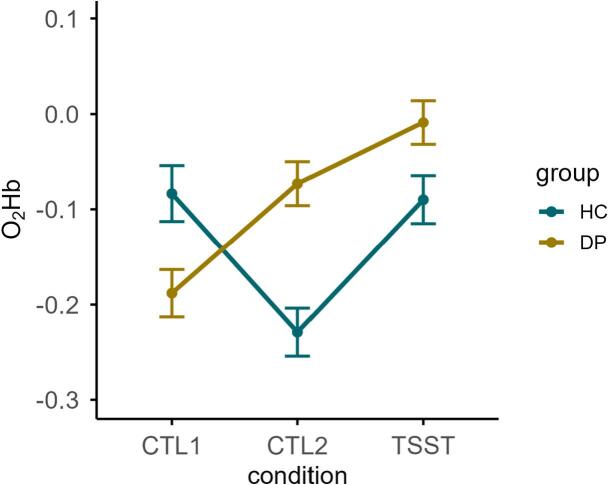
Line plot of the raw data of anticipatory (baseline) fNIRS data (O_2_Hb) dependent on condition (CTL1 = control task 1, i.e. reading numbers; CTL2 = control task 2, i.e. mental arithmetics without TSST panel; TSST = arithmetic task of the TSST, i.e. mental arithmetics with TSST panel) for two groups: HC (healthy controls) and DP (patients with depression) during the rest phase (window 1 in figure 2). Please note that the data is averaged over all ROIs as it illustrates the significant interaction of condition and group. Lines depict the mean cui-corrected O_2_Hb-signals for each group, error bars represent +/- 1 standard error of the mean (SE).

Benjamini-Hochberg corrected pairwise comparisons of the interaction of condition and group indicated no significant (*p_corr_* < 0.05) differences between the groups at any time point but when considering differences between consecutive conditions, we observed significant increases in O_2_Hb between CTL2 and the arithmetic task of the TSST in HC (mean difference = -0.139, CI [-0.244;–0.033], *SE* = 0.054, *p* < 0.05) (see Fig. 5).

There was further a significant interaction of ROI and group for O_2_Hb (*F*(3.375, 469.150) = 2.647, *p* < 0.05, $\eta_{p}^{2}$ = 0.019, HHb: *F*(2.722, 370.182) = 0.909, *p* = 0.429, $\eta_{p}^{2}$ = 0.007), as well as a significant main effect of ROI for O_2_Hb (*F*(3.375, 469.150) = 4.251, *p* < 0.01, $\eta_{p}^{2}$ = 0.030, HHb*: F*(2.722, 370.182) = 2.677, *p* = 0.052, $\eta_{p}^{2}$ = 0.019).

No significant interaction with age occurred but a main effect of age for O_2_Hb (*F*(1, 139) = 4.988, *p* < 0.05, $\eta_{p}^{2}$ = 0.035, HHb: *F*(1, 136) = 3.801, *p* = 0.053, $\eta_{p}^{2}$ = 0.027).

**Fig. 6:**
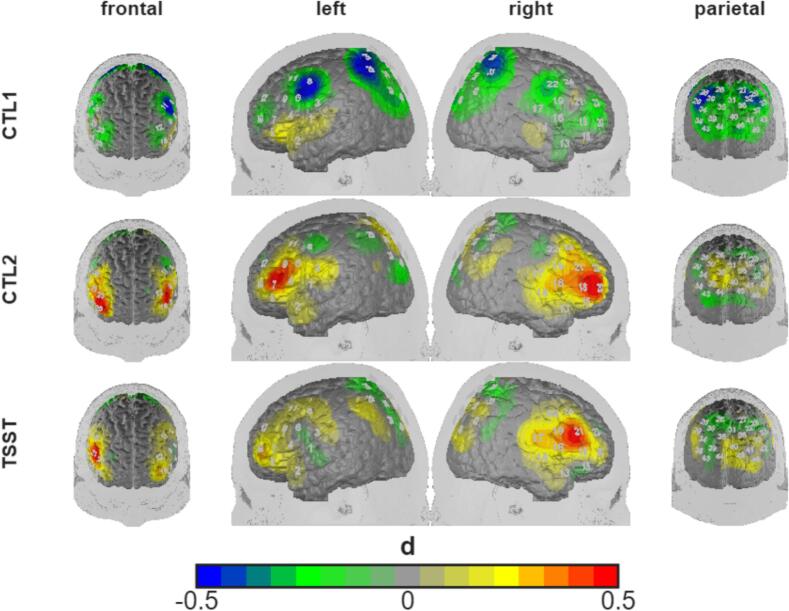
Differences in cortical oxygenation (O_2_Hb) between HC (healthy controls) and DP (patients with depression) dependent on condition (CTL1 = control task 1, i.e. reading numbers; CTL2 = control task 2, i.e. mental arithmetics without TSST panel; TSST = arithmetic task of the TSST, i.e. mental arithmetics with TSST panel) prior to the beginning of the trials (window 1 in figure 2). Warm colors indicate increased O_2_Hb-levels in DP as compared to HC; cool colors vice versa. Differences are depicted in Cohen's *d*. Numbers indicate fNIRS channels. left IFG = channel 6, 9, 7; right IFG = channel 18, 19, 21; left DLPFC = channel 10, 11, 12; right DLPFC = 20, 23, 24; SAC = channel 25, 26, 27, 28, 30, 31, 32, 35, 36.

For a visualization of the time series of window 1 see supplementary material S10. For brainmaps see Fig. 6.

### 3.5 Exploratory analyses

Finally, we examined the relationship between neural activation during window 1 and stress-related measures (see Table 2). Activation levels across all ROIs were significantly correlated, with moderate to strong positive associations ranging from *r*(141) = 0.288, *p* < 0.001 (right IFG with SAC) to *r*(141) = 0.799, *p* < 0.001 (left DLPFC with right DLPFC). With respect to physiological stress parameters, neural activation in none of the ROIs was significantly associated with salivary cortisol levels measured 15 min after the TSST (peak of cortisol levels in both groups), nor with heart rates during the arithmetic task of the TSST. Notably, these physiological indicators were also not significantly related to subjective stress, negative affect, or state rumination.

**Table 2:** Two-sided Pearson correlations investigating the association of neural activation prior to task onset in the different ROIs and different physiological and behavioral variables.

	left DLPFC	right IFG	right DLPFC	SAC	cortisol	heart rate	NA	SRSRQ	stress
left IFG	0.657^***^	0.478^***^	0.483^***^	0.311^***^	0.088	−0.139	0.082	0.117	0.080
left DLPFC		0.465^***^	0.799^***^	0.575^***^	−0.071	−0.138	0.140	0.215*	0.137
right IFG			0.522^***^	0.288^***^	−0.090	−0.165	0.159	0.266^**^	0.144
right DLPFC				0.568^***^	−0.026	−0.135	0.086	0.208*	0.090
SAC					−0.018	−0.054	0.001	0.015	0.070
cortisol						0.061	−0.060	−0.074	−0.105
heart rate							0.078	−0.018	−0.019
NA								0.753^***^	0.670^***^
SRSRQ									0.513^***^

**Note.** Numbers represent correlation coefficients, asterisks represent uncorrected significance levels. * = *p* < 0.05, ** = *p* < 0.01, *** = *p* < 0.001, IFG = Inferior Frontal Gyrus, DLPFC = Dorsolateral Prefrontal Cortex, SAC = Somatosensory Association Cortex, heart rate = heart rate in beats per minute during the arithmetic task of the TSST, cortisol = salivary cortisol 15 min after the TSST (peak of cortisol levels in both groups), NA = negative affect scale of the PANAS 0 min after the TSST, SRSRQ = Stress-Reactive State Rumination Questionnaire after resting-state 2, Stress = subjective stress assessed using a Visual Analogue Scale 0 min after the TSST. Note that all of the significant correlations remain significant at *p* < 0.05 when correcting row-wise for multiple comparisons using the Benjamini-Hochberg procedure.

In contrast, state rumination assessed after the second resting-state (SRSRQ scores) was positively associated with neural activation in the left DLPFC (*r*(141) = 0.215, *p* < 0.05), right IFG (*r*(141) = 0.266, *p* < 0.01), and right DLPFC (*r*(141) = 0.208, *p* < 0.05). Correlation coefficients ranged from 0.208 to 0.266, indicating that greater neural activation prior to task onset was associated with higher levels of state rumination following the stressor. Moreover, state rumination was strongly associated with negative affect immediately after the TSST (*r*(141) = 0.753, *p* < 0.001) and with subjective stress following the TSST (*r*(141) = 0.513, *p* < 0.001). Finally, subjective stress and negative affect were themselves strongly correlated (*r*(141) = 0.670, *p* < 0.001), indicating higher negative affect in case of higher subjective stress.

## 4 Discussion

While much research has focused on cognitive, affective, and neurophysiological processes during stress exposure, the Neurocognitive Framework for Regulation Expectation (NFRE; De Raedt and Hooley, 2016) emphasizes that processes occurring prior to stressor confrontation critically shape the subsequent stress response. In healthy individuals, this anticipatory phase is thought to involve proactive recruitment of prefrontal control regions that support adaptive regulation. In contrast, depression is associated with alterations in these processes, potentially reflecting dysfunctional expectations about coping ability and resulting in maladaptive patterns of neural engagement. Such aberrant anticipation may contribute to dysregulated stress responses and is therefore highly relevant for understanding underlying neurocognitive mechanisms. Although reduced prefrontal activation under stress is a robust finding in patients with depression, neural activity preceding stress exposure may systematically bias estimates of task-related activation.

In the present study, we pooled data from two prior investigations employing comparable experimental paradigms to enhance statistical power and to assess how anticipation-related neural activity influences stress-related brain activation (Laicher et al., 2023, Rosenbaum et al., 2021, Rosenbaum et al., 2024). Both studies included two non-stressful control tasks (number reading and mental arithmetic without time or social pressure) as well as the arithmetic task of the TSST, which incorporates both time pressure and increased social-evaluative threat compared to the control tasks. For fNIRS data acquisition, each of the three tasks included a total of six trials with 40 s of task performance and a subsequent 20 s rest period, which concurrently served as the period prior to the following trial.

We initially evaluated the stress response and found significant increases in subjective stress, negative affect, and cortisol levels following the TSST, indicating effective stress induction across behavioral and physiological variables.

We then analyzed neural activation in response to the TSST, initially applying a standard analysis approach in which event-related averages were calculated using a baseline correction based on the five seconds immediately preceding each trial onset. Consistent with prior findings this analysis revealed a well-known pattern of prefrontal hypoactivation in DP under stress (Int-Veen et al., 2023, Pizzagalli and Roberts, 2022, Rosenbaum et al., 2018a, Rosenbaum et al., 2018b, Rosenbaum et al., 2021, Rosenbaum et al., 2024). Specifically, while neural activation during the trials did not differ between groups during the control tasks, DP exhibited less pronounced increases in cortical oxygenation during the arithmetic task of the TSST compared to HC.

When correlating brain activation during the arithmetic task of the TSST with subjective and physiological stress in a supplementary analysis (see supplementary material S6 Table S6.2), we observed significant negative correlations between the IFG, DLPFC and SAC with physiological and subjective stress responses.

Crucially, however, this pattern of hypoactivation in DP was reversed when we focused on neural activity preceding task onset. When event-related averages were computed for the 15 s prior to each trial, again significant group-by-condition differences emerged. This time, however, Benjamini-Hochberg-corrected pairwise comparisons indicated that DP showed significant increases in cortical oxygenation from non-stressful control conditions to the TSST. In contrast, HC showed no changes between the first control task and the arithmetic task of the TSST and even decreases between the easier and the more difficult control task. This divergent pattern suggests fundamentally different temporal dynamics of neural engagement prior to task execution between both groups. Note that there were no overall ROI-specific effects, but this pattern was apparent in all regions, namely the bilateral IFG, DLPFC and SAC.

When we subsequently applied a baseline correction that accounted for this activation prior to the beginning of each trial by again using the interval from −20 to −15 s prior to trial onset as baseline and averaging from 0 to 40 s after task onset, the previously observed group differences in stress-related brain activation disappeared. Using this forward-shifted baseline, there were no longer significant group-by-condition interactions, and hypoactivation in DP during stress was no longer evident.

When using this “corrected” stress-related brain activation to correlate brain activation during the TSST with subjective and physiological stress (see supplementary material S6 Table S6.1), we observed far fewer significant correlations, complementing the absent group differences.

These findings suggest that DP might recruit task-relevant brain regions substantially earlier than HC. From this perspective, the task-related prefrontal hypoactivation commonly observed in DP under stress may not indicate a failure to engage cognitive control mechanisms per se, but rather be affected by altered activation prior to the stress response.

Generally, this is in line with the Neurocognitive Framework for Regulation Expectation (NFRE; De Raedt and Hooley, 2016), however following this framework a reduced recruitment of the DLPFC in anticipation of a stressor would have been expected.

The TSST is a widely used paradigm to investigate the behavioral, physiological and neural stress response (Allen et al., 2017, Kudielka et al., 2007) and especially also cognitive and physiological effects of stressor anticipation (Gaab et al., 2005, Nasso et al., 2019, Pulopulos et al., 2020, Pulopulos et al., 2022). In the current study, we adapted the arithmetic task of the TSST to a block design consisting of six trials, each including 40 s of stressor exposure followed by a 20-second pause. As a result, the 20-second “anticipation” period preceding each of the last five trials also functioned as the post-trial interval of the previous trial. While this design allows us to assess anticipatory processes prior to stressor exposure, it does not permit us to disentangle whether increased anticipatory brain activation reflects thoughts about the upcoming trial, the preceding trial, or a combination of both. At the psychological level, processing of the preceding trial may be related to ruminative thinking, that is, repetitive and self-referential negative thoughts about past performance or perceived failure (Nolen-Hoeksema et al., 2008, Watkins and Roberts, 2020). Elevated anticipatory activation may indicate that DP engaged more strongly with the upcoming task in advance, which could reflect increased worry, threat monitoring, increased self-focus, or internally focused processing rather than efficient preparation. This means that cognitive and neural resources might be consumed by ruminative thinking and therefore attentional control cannot be redirected efficiently to the task, which ultimately might lead to impaired performance.

This is further supported by significant correlations of neural activation prior to task onset with state rumination. Note, however, that separating the effects of rumination from those of depressive symptomatology remains inherently challenging. Because ruminative thinking (in the correlation analysis specifically state rumination as assessed using the SRSRQ) and depressive symptomatology (BDI-II) have a substantial amount of shared variance (see Int-Veen et al., 2023), the results may also reflect a broader association with general symptom severity or negative affectivity. This concern is further supported by the moderate to strong intercorrelations observed among self-report measures, particularly the strong association between state rumination and negative affect following the TSST. Additionally, when correlations are computed separately for HC and DP, those involving state rumination are largely no longer significant in the DP group (see supplementary material S11). Note, however, that these correlation analyses were exploratory in nature. The primary focus of the study lay on the neural effects, whereas the correlations were conducted to examine whether the heightened activation preceding arithmetic trials of the TSST might be associated with behavioral or physiological measures.

It is worth noting that participants knew that the next calculation trial would begin with a different number, which limited their ability to prepare extensively; nevertheless, the task structure remained the same. These effects cannot be disentangled in the current analysis, but they provide an opportunity for future studies. For example, one might isolate true anticipatory effects by analyzing only the first trial. Unfortunately, we were unable to conduct the suggested analysis due to limitations in the available data arising from the specific experimental procedure, namely differences across participants in the pre-trial interval of the first trial.

Apart from the investigation of the anticipation of the next trial, it would further be interesting to investigate the (neuro-)physiological response during the actual anticipation of the TSST, more specifically the 5 min preparation phase prior to the job interview. While the TSST has been widely used to investigate cognitive and physiological effects of stressor anticipation (Gaab et al., 2005, Nasso et al., 2019, Pulopulos et al., 2020, Pulopulos et al., 2022), future studies would benefit from experimental designs that more clearly disentangle anticipatory and post-event neural processes, such as event-related paradigms with jittered inter-trial intervals, extended baseline periods, or explicit anticipation cues followed by sufficiently long delay phases. In addition, when using a TSST adaptation similar to the one employed here, incorporating trial-based subjective ratings could help clarify whether participants are primarily focused on the upcoming or the preceding trial, thereby providing a more direct assessment of the cognitive processes underlying anticipatory neural activity.

When interpreting the aforementioned results, several further limitations have to be noted. Given that the DP group in study 2 was, on average, approximately 5 years older than the HC group, there was also an age difference of approximately 4 years in the resulting total sample. To investigate the potential effects of this age difference, the analyses were conducted with and without age as a covariate. The results suggest that the effects were not influenced by the age differences and appear to be robust, potentially because age differences within this adult range (27 to 32 years) may be less pronounced than at younger or older stages of life.

Another limitation of the current investigation is that we did not include a non-mathematical control task. Therefore, we cannot determine whether the observed effects reflect general mechanisms or are specific to mental arithmetic tasks.

Further, an important consideration is that the CTL2 condition may have partially attenuated the incremental stress effect of the subsequent TSST arithmetic component. Specifically, CTL2 involved a similar serial subtraction format and included corrective feedback, which may have introduced a mild form of performance monitoring and reduced task novelty. Although CTL2 lacked the defining characteristics of the Trier Social Stress Test — in particular, strong social-evaluative threat and uncontrollability — this prior exposure may nonetheless have familiarized participants with the task structure and slightly diminished the stress impact of the later arithmetic task and therefore further influenced expectancy and anticipation.

Lastly, increasing evidence suggests that anxiety and depression are associated with partially distinct prefrontal activation patterns. For instance, recent fNIRS work demonstrates that DLPFC oxygenation during emotional autobiographical memory processing can reliably differentiate between anxiety and depression, highlighting disorder-specific neural signatures (Wang et al., 2025). Moreover, studies on implicit emotion regulation indicate that both major depressive disorder and generalized anxiety disorder show reduced DLPFC activation, yet differ in associated prefrontal dynamics and functional correlates, such as orbitofrontal involvement and links to symptom dimensions (Wang et al., 2024). Together, these findings suggest that comorbid or subclinical anxiety symptoms might influence the effects in the present study.

To further examine this possibility, we conducted a reanalysis using LSAS-based groups (Liebowitz Social Anxiety Scale) with a cutoff score of 30 to distinguish between individuals with elevated versus low social anxiety (Rytwinski et al., 2009). Unfortunately, we did not assess general symptoms of anxiety. This analysis did not reveal any significant effects of LSAS group in any of the three time windows (see supplementary material S12). Future research should therefore include dimensional assessments of anxiety to better disentangle disorder-specific contributions to anticipatory and stress-related prefrontal activation.

In conclusion, our findings underscore the critical importance of baseline selection when interpreting group differences in task-related neural activation. Apparent hypoactivity could, at least partially, reflect relatively higher baseline activity in DP rather than reduced task-related activation per se.

## 5 Declaration of generative AI use

During the preparation of this work the authors used ChatGPT (GPT-5.2) to assist with language editing and grammar checking. After using this tool, the authors reviewed and edited the content as needed and take full responsibility for the content of the published article.

## CRediT authorship contribution statement

**Isabell Int-Veen:** Writing – review & editing, Writing – original draft, Visualization, Investigation, Formal analysis, Data curation. **Ann-Christine Ehlis:** Writing – review & editing, Writing – original draft, Validation, Supervision, Resources, Project administration. **Agnes Kroczek:** Writing – review & editing, Writing – original draft. **Hendrik Laicher:** Writing – review & editing, Writing – original draft. **Andreas J. Fallgatter:** Writing – review & editing, Writing – original draft, Validation, Resources, Methodology. **David Rosenbaum:** Writing – review & editing, Writing – original draft, Validation, Supervision, Project administration, Methodology, Funding acquisition, Conceptualization.

## Funding

This research was partly supported by fortüne funding program at the University of Tuebingen (grant no 2570–1-0).

## Declaration of Competing Interest

The authors declare that they have no known competing financial interests or personal relationships that could have appeared to influence the work reported in this paper.

## Data Availability

Data will be made available on request.

## Appendix A Supplementary data

Supplementary Data 1 The supplementary data contain details regarding the study procedures, data preprocessing, and additional analyses.
