# Supplementary material for: Stress-related hypofrontality in depression and its relation to altered activation prior to the stress response
Source: Neuroimage Clin. 2026 May 29;51:104019. doi: 10.1016/j.nicl.2026.104019 (PMC13277536; doi:10.1016/j.nicl.2026.104019)
Supplement: Supplementary Data 1 — The supplementary data contain details regarding the study procedures, data preprocessing, and additional analyses. [file mmc1.pdf]

Supplementary Material to:

Stress-related hypofrontality in depression and its relation to altered activation prior to the stress response

Isabell Int-Veen[1,2], Ann-Christine Ehlis[1,2,3], Agnes Kroczek[1], Hendrik Laicher[1,2], Andreas J. Fallgatter[1,2,3], David Rosenbaum[1]

[1] Department of Psychiatry and Psychotherapy, Tübingen Center for Mental Health (TüCMH), University of Tübingen, Tübingen, Germany
[2] German Center for Mental Health, partner site Tübingen, Germany
[3] LEAD Graduate School & Research Network, University of Tuebingen, Tuebingen, Germany

Corresponding Author:
Isabell Int-Veen
Calwerstraße 14
72076 Tübingen
Germany

Supplementary material S1: Consort diagrams of the studies

Study 1:

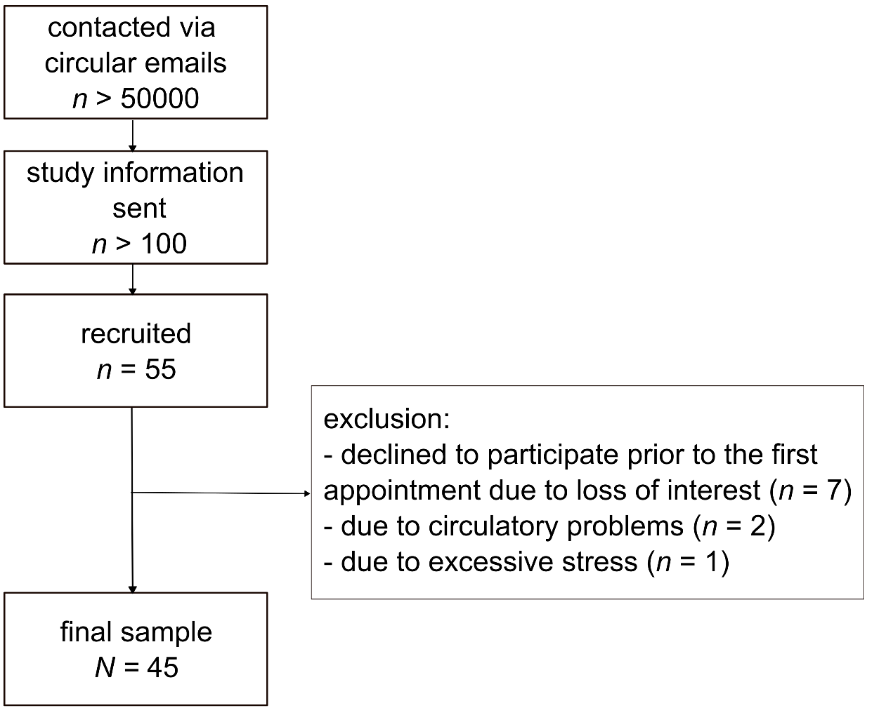

Study 2:

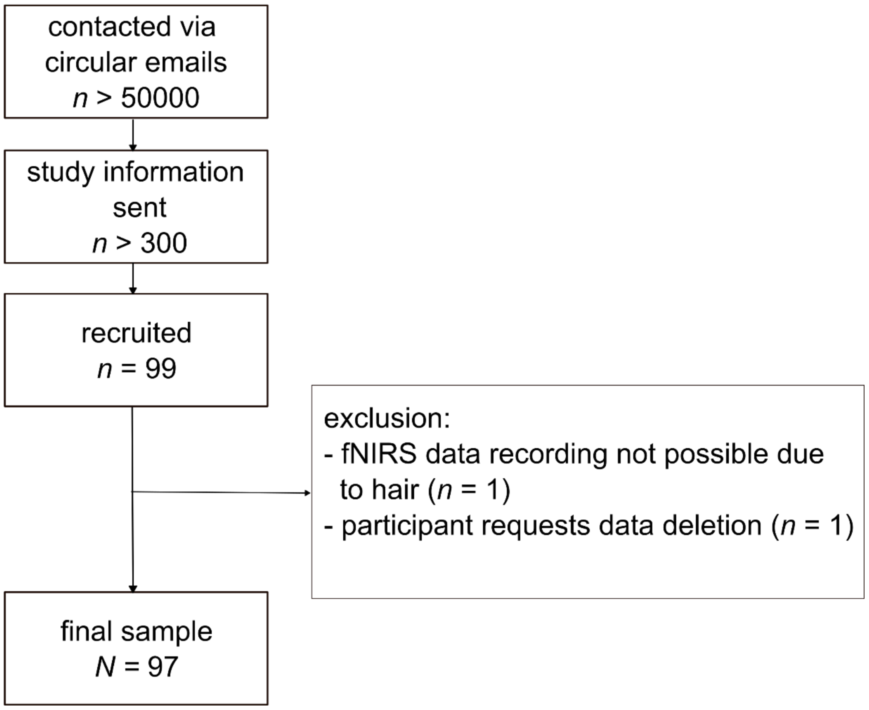

Note. study 1 = Rosenbaum et al., 2021; study 2 = Laicher et al., 2023; Rosenbaum et al., 2024.

Supplementary material S2: Inclusion and exclusion criteria of the studies

Inclusion criteria:

study 1	study 2
18-40 years	18-60 years
normal vision (or appropriate correction)	normal vision (or appropriate correction)
German as native language or very good knowledge of German	German as native language or very good knowledge of German
right-handedness	right-handedness

Exclusion criteria:

study 1	study 2
diabetes mellitus	diabetes mellitus
renal insufficiency	renal insufficiency
untreated hypertension	untreated hypertension
history of traumatic brain injury	history of traumatic brain injury
cardiac arrhythmia	cardiac arrhythmia
Cushing Syndrome	Cushing Syndrome
adrenal insufficiency	adrenal insufficiency
For HC: any acute psychiatric or neurological disorder ((including any anomalies in the Structured Clinical Interview (SCID); First et al., 2015)	For HC: any acute psychiatric or neurological disorder ((including any anomalies in the Structured Clinical Interview (SCID); First et al., 2015)
For DP: other primary mental disorders than ICD-10 diagnoses F32.x, F34.1, or F33.x as well as acute suicidal tendencies, extremely severe depressive symptoms (BDI-II > 50), emotional instability as assessed by the treating psychologist, and previous decompensation under social stress	For DP: other primary mental disorders than ICD-10 diagnoses F32.x, F34.1, or F33.x as well as acute suicidal tendencies, extremely severe depressive symptoms (BDI-II > 50), emotional instability as assessed by the treating psychologist, and previous decompensation under social stress
smokers >3 cigarettes per day	
in case of women: pregnancy	in case of women: pregnancy

Note. study 1 = Rosenbaum et al., 2021; study 2 = Laicher et al., 2023; Rosenbaum et al., 2024, BDI-II = Beck's Depression Inventory II, HC = healthy controls, DP = patients with depression. The exclusion of participants with a BDI score > 50, the exclusion of individuals with acute suicidality, clinically assessed emotional instability, and a history of decompensation under social stress was implemented as a safety measure in accordance with the requirements of the ethics committee. Specifically, the committee requested that we minimize the risk of potential decompensation during exposure to the stress induction procedure (TSST). Thus, this exclusion criterion was not theoretically motivated but rather ethically driven to ensure participant safety.

Supplementary material S3: Items of the Stress-Reactive State Rumination Questionnaire

Scoring: The items are rated on a 5-point Likert-Scale (1 = "not at all", 2 = "almost not", 3 = "a little", 4 = "often" and 5 = "very often"). Please note that items 7 and 13 are reverse-coded. The sum-score is consequently calculated as follows: item1 + item2 + item3 + item4 + item5 + item6 + (6 - item7) + item8 + item9 + item10 + item11 + item12 + (6 - item13) + item14 + item15 + item16 + item17 + item18

Item	German translation	English translation	Dutch translation
	Im Folgenden werden Ihnen Fragen zur letzten Ruhephase gestellt. Wir bitten Sie anzugeben, inwiefern die folgenden Aussagen über Ihr Erleben während dieser Zeit übereinstimmen. Sie können hierzu die folgenden Einschätzungen abgeben: „gar nicht“, „fast nicht“, „ein wenig“, „oft“ und „sehr oft“.	In the following, you will be asked questions about the last rest period. We ask you to indicate to what extent the following statements about your experience during this time agree with each other. You can give the following ratings: "not at all", "almost not", "a little", "often" and "very often".	In deze vragenlijst wordt u gevraagd om aan te geven hoe u in de afgelopen rustperiode hebt nagedacht. Lees de onderstaande uitspraken door en geef aan in welke mate ze voor u in de afgelopen rustperiode van toepassing waren
1[2]	Ich dachte immer wieder an meine Probleme.	I kept thinking about my problems.	Ik bleef aan mijn problemen denken.
2[2]	Ich verharrte im Denken an Dinge, die mich beunruhigen.	I persisted in thinking about things that worried me.	Ik bleef denken aan dingen die mij dwarszitten.
3[2]	Meine Gedanken wiederholten sich, ohne dass ich zu einer Lösung kam.	My thoughts repeated themselves without me coming to a solution.	Ik dacht over veel problemen na zonder ze op te lossen.
4[4]	Ich verlor mich in meinen negativen Gedanken.	I lost myself in my negative thoughts.	Ik raakte verloren in mijn negatieve gedachten.
5[3]	Ich konnte meine Gedanken nur mühsam festhalten.	I had difficulty holding on to my thoughts.	Ik had moeite om mijn gedachten vast te houden.
6[4]	Ich konnte mich nicht von meinen negativen Gedanken lösen.	I could not get away from my negative thoughts.	Ik kon mijn negatieve gedachten niet loslaten.
7[4]	Ich war bei der Sache.	My mind was on the matter at hand.	Ik was aanwezig.
8[1]	Ich dachte darüber nach, warum ich mich in bestimmten Situationen falsch verhalten habe.	I thought about why I was behaving wrongly in certain situations.	Ik dacht over waarom ik fout gehandeld heb in bepaalde situaties.
9[1]	Ich fragte mich, warum ich Probleme habe, die andere nicht haben.	I wondered why I had problems that others didn't.	Ik vroeg mij af waarom ik problemen heb die andere mensen niet hebben.
10[1]	Ich fragte mich, womit ich meine momentane Lebenssituation verdient habe.	I asked myself what I did to deserve my current situation in life.	Ik vroeg me af waar ik mijn huidige levenssituatie aan verdiend heb.

11[1]	Ich dachte darüber nach, warum ich die Dinge nicht besser in den Griff bekomme.	I thought why can't I handle things better	Ik vroeg me af waarom ik dingen niet beter aankan.
12[1]	Ich dachte an all meine Defizite und Misserfolge, Macken und Fehler.	I thought about all my shortcomings and failures, quirks and mistakes.	Ik dacht aan al mijn gebreken, tekorten, fouten, vergissingen.
13[4]	Ich konnte flexibel zwischen meinen Gedanken hin und her schalten.	I had the flexibility to switch back and forth between my thoughts.	Ik kon flexibel schakelen tussen mijn gedachten.
14[1]	Ich dachte an vergangene Situationen, die ich bereue.	I thought of past situations that I regret.	Ik dacht aan voorbije situaties waar ik spijt van heb.
15[1]	Ich machte mir Selbstvorwürfe.	I blamed myself.	Ik gaf mezelf de schuld.
16[4]	Ich verlor mich in Gedanken an Vergangenes.	I got lost in thoughts of things past.	Ik verdwaalde in gedachten over het verleden.
17[4]	Ich war von meinen Problemen und Sorgen stark vereinnahmt.	I was strongly absorbed by my problems and worries.	Mijn gedachten en problemen namen mij volledig in beslag.
18[2]	Meine negativen Gedanken ließen mich nicht los.	My negative thoughts did not let me go.	Ik kon mijn negatieve gedachten niet loslaten.

[1] item adapted from the Ruminative Response Scale (RRS; Nolen-Hoeksema & Morrow, 1991)

[2] item adapted from the Perseverative Thinking Questionnaire (PTQ; Ehring et al., 2011)

[3] item adapted from the Amsterdam Resting State Questionnaire (ARSQ; Diaz et al., 2013)

[4] item adapted from the rumination questionnaire by de Jong-Meyer and colleagues (2009).

Supplementary material S4: Details on recording and preprocessing of cortisol and ECG data

Cortisol. Saliva was collected using Salivettes (Sarstedt AG & Co., REF 51.1534.500). Participants were instructed to gently chew on the cotton swab for two minutes to ensure sufficient saliva absorption and then return the swab to the tube without touching it. Approximately one hour prior to saliva collection, participants were instructed to refrain from eating or drinking to prevent dilution or contamination of the sample. Samples were stored at -20 °C until further processing. After data collection from all participants was completed, the Salivettes were centrifuged for 2 minutes at 1000 g. Cortisol concentrations were determined using an enzyme-linked immunosorbent assay (IBL International, Cortisol ELISA, REF RE52611) in accordance with the manufacturer's instructions. As saliva samples were collected throughout the day and cortisol levels are subject to circadian variation, the effect of time of day was regressed out of the cortisol data, and the resulting residuals were used for statistical analyses.

Heart rate. Heart rate was assessed using a single-channel electrocardiogram (ECG). After disinfection of the relevant skin areas, two standard Ag/AgCl ring electrodes (8 mm diameter) were attached with conductive EEG paste, one positioned above the right clavicle and the other below the left costal arch. An additional ring electrode was placed on the neck as a reference. The ECG signal was recorded using a BrainAmp ExG amplifier and BrainVision Recorder software (Brain Products, Munich, Germany) at a sampling rate of 1000 Hz. Data preprocessing and analysis were performed using BrainVision Analyzer 2.1 and MATLAB 2017a. Preprocessing included band-passfanti filtering with a low cutoff at 1 Hz (time constant = 0.0318 s, 48 dB/oct) and a high cutoff at 30 Hz (48 dB/oct), following established guidelines (Kligfield et al., 2007). Data from one participant were heavily contaminated by power-line artifacts; therefore, an additional 50 Hz notch filter was applied. For each task separately, R-peaks were detected using the pulse artifact correction tool, and heart rate was calculated as the mean interval between successive R-peaks, expressed in beats per minute.

Supplementary material S5: Placement of the fNIRS probesets and Regions of Interest

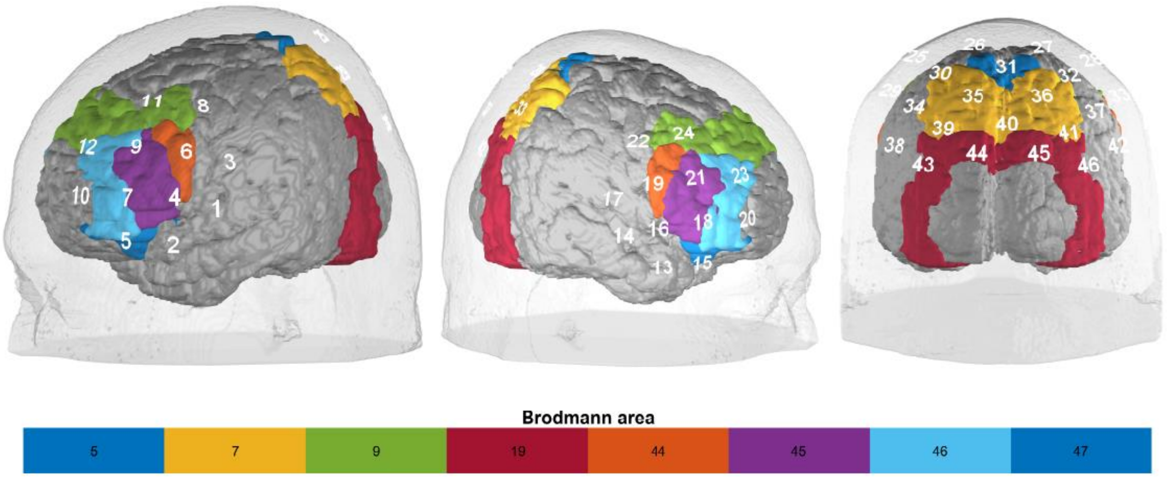

Figure S5. Placement of the three probesets: two frontal (left and right) and one parietal. Note that the corresponding numbers represent the channels (located midway between emitter and detector). Scalp-brain correspondence was estimated based on Okamoto et al. (2004), Okamoto and Dan (2005) as well as Singh et al. (2005). IFG = Inferior Frontal Gyrus, DLPFC = Dorsolateral Prefrontal Cortex, SAC = Somatosensory Association Cortex.

Table S5: Assignment of probeset channels to Regions of Interest

**ROI**	**channels**
left IFG	6 9 7
right IFG	19 21 18
left DLPFC	11 12 10
right DLPFC	24 23 20
SAC	25 26 27 28 30 31 32 35 36

Note. IFG = Inferior Frontal Gyrus, DLPFC = Dorsolateral Prefrontal Cortex, SAC = Somatosensory Association Cortex.

Supplementary material S6: Correlations of task-related neural activation (window 2 and 3) with subjective and physiological stress responses

window 2 (task-related brain activation with baseline accounting for different baselines)

Table S6.1. Two-sided Pearson correlations investigating the association of window 2 neural activation in the different ROIs (left IFG, left DLPFC, right IFG, right DLPFC, SAC) and different physiological and behavioral variables.

	left DLPFC	right IFG	right DLPFC	SAC	cortisol	heart rate	NA	SRSRQ	stress
left IFG	**.608*** **	**.543*** **	**.501*** **	**.324*** **	-.195*	-.115	-.162	-.108	-.064
left DLPFC		**.456*** **	**.679*** **	**.492*** **	-.170	-.109	-.187*	-.118	-.083
right IFG			**.582*** **	**.210***	-.173	-.186	-.027	.052	.047
right DLPFC				**.543*** **	-.140	-.081	-.145	-.022	-.070
SAC					.018	-.101	-.151	-.209*	-.097
cortisol						.061	-.060	-.074	-.105
heart rate							.078	-.018	-.019
NA								**.753*** **	**-670*** **
SRSRQ									**.513*** **

Note. Numbers represent correlation coefficients, asterisks represent uncorrected significance levels. * = p < .05, ** = p < .01, *** = p < .001, IFG = Inferior Frontal Gyrus, DLPFC = Dorsolateral Prefrontal Cortex, SAC = Somatosensory Association Cortex, heart rate = heart rate in beats per minute during the arithmetic task of the TSST, cortisol = salivary cortisol 15 min after the TSST, NA = negative affect scale of the PANAS 0 min after the TSST, SRSRQ = Stress-Reactive State Rumination Questionnaire after resting-state 2, Stress = subjective stress assessed using a Visual Analogue Scale 0 min after the TSST. Bold correlations remain significant at p < .05 when correcting row-wise for multiple comparisons using the Benjamini-Hochberg procedure.

window 3 (task-related brain activation with standard baseline)

Table S6.2. Two-sided Pearson correlations investigating the association of window 3 neural activation in the different ROIs (left IFG, left DLPFC, right IFG, right DLPFC, SAC) and different physiological and behavioral variables.

	left DLPFC	right IFG	right DLPFC	SAC	cortisol	heart rate	NA	SRSRQ	stress
left IFG	**.500*****	**.494*****	**.354*****	**.183*****	-.198*	.095	-.151	-.196*	-.117
left DLPFC		**.467*****	**.654*****	**.585*****	-.022	.105	**-.332*****	**-.387*****	**-.271****
right IFG			**.553*****	**.251****	-.080	.055	**-.222****	**-.298*****	**-.195***
right DLPFC				**.517*****	-.074	.084	**-.218****	**-.259****	**-.206***
SAC					.069	-.035	**-.184***	**-.271****	**-.213***
cortisol						.061	-.060	-.074	-.105
heart rate							.078	-.018	-.019
NA								**.753*****	**.670*****
SRSRQ									**.513*****

Note. Numbers represent correlation coefficients, asterisks represent uncorrected significance levels. * = p < .05, ** = p < .01, *** = p < .001, IFG = Inferior Frontal Gyrus, DLPFC = Dorsolateral Prefrontal Cortex, SAC = Somatosensory Association Cortex, heart rate = heart rate in beats per minute during the arithmetic task of the TSST, cortisol = salivary cortisol 15 min after the TSST, NA = negative affect scale of the PANAS 0 min after the TSST, SRSRQ = Stress-Reactive State Rumination Questionnaire after resting-state 2, Stress = subjective stress assessed using a Visual Analogue Scale 0 min after the TSST. The gray shading of the table cell indicates a significant correlation in window 2 (see table S6.1). Bold correlations remain significant at p < .05 when correcting row-wise for multiple comparisons using the Benjamini-Hochberg procedure.

Supplementary material S7: Results of fNIRS analyses using age as a covariate

Stress-related brain activation.

To investigate stress-related brain activation, we fitted a rmANOVA to the fNIRS data during the tasks using the standard baseline correction just before the trial began (window 3 in figure 2). As a result, we observed a significant interaction of condition and group for O_2Hb, $F(2, 280) = 5.790$, $p < .01$, $\eta_p^2 = .040$ (HHb: $F(2, 276) = 0.861$, $p = .424$, $\eta_p^2 = .006$). Benjamini-Hochberg-corrected pairwise comparisons of this interaction yielded significant ($p_{corr} < .05$) differences between HC and DP only during the TSST (mean difference = -0.202, CI [-0.340; -0.064], $SE = 0.070$, $p < .01$) and while cortical oxygenation increased between all three conditions significantly for HC (control task 1 to control task 2: mean difference = -0.115, CI [-0.286; -0.024], $SE = 0.066$, $p < .05$; control task 2 to TSST: mean difference = -0.192, CI [-0.317; -0.068], $SE = 0.066$, $p < .05$), there were no differences between conditions in DP. Further, we found significant main effects of ROI in O_2Hb, $F(3.381, 473.303) = 11.566$, $p < .001$, $\eta_p^2 = .076$, as well as HHb, $F(3.301, 455.515) = 3.172$, $p < .05$, and main effects of condition in O_2Hb, $F(2, 280) = 9.788$, $p < .001$, $\eta_p^2 = .065$, and HHb, $F(2, 276) = 8.903$, $p < .001$, $\eta_p^2 = .061$, indicating general increases in cortical oxygenation from the control tasks to the TSST.

When we fitted a rmANOVA using the fNIRS data but using the baseline correction to account for activation prior to task onset (window 2 in figure 2), we observed no significant interaction of condition and group (O_2Hb: $F(2, 280) = 1.879$, $p = .155$, $\eta_p^2 = .013$, HHb: $F(2, 276) = 1.151$, $p = .318$, $\eta_p^2 = .008$), only a significant interaction of condition and ROI (O_2Hb: $F(6.497, 909.604) = 15.576$, $p < .001$, $\eta_p^2 = .100$, HHb: $F(6.090, 840.354) = 9.196$, $p < .001$, $\eta_p^2 = .62$) and significant lower-order main effects of ROI (O_2Hb: $F(3.440, 481.565) = 64.392$, $p < .001$, $\eta_p^2 = .315$, HHb: $F(3.340, 460.964) = 3.611$, $p < .05$, $\eta_p^2 = .026$) and condition (O_2Hb: $F(2, 280) = 36.965$, $p < .001$, $\eta_p^2 = .209$, HHb: $F(2, 276) = 39.927$, $p < .001$, $\eta_p^2 = .224$) again, indicating stress-related increases in brain activation.

Anticipation-related brain activation.

Fitting a rmANOVA to investigate the neural activation in both groups prior to the onset of the trial, there was a significant interaction of condition and group in O_2Hb, $F(2, 280) = 5.892$, $p < .01$, $\eta_p^2 = .040$ (HHb: $F(2, 274) = 1.406$, $p = .247$, $\eta_p^2 = .010$), a significant interaction of condition and ROI in O_2Hb, $F(6.027, 843.808) = 8.174$, $p < .001$, $\eta_p^2 = .055$ (HHb: $F(6.059, 830.150) = 1.914$, $p = .075$, $\eta_p^2 = .014$), as well as a significant interaction of ROI and group in O_2Hb, $F(3.370, 471.745) = 3.358$, $p < .05$, $\eta_p^2 = .023$ (HHb: $F(2.742, 375.640) = 1.409$, $p = .241$, $\eta_p^2 = .010$).

Benjamini-Hochberg-corrected pairwise comparisons of the interaction of condition and group indicated that DP exhibited significantly higher (p_{corr} < .05) cortical oxygenation than HC during control task 2 (mean difference = -0.156, CI [-0.274; -0.038], *SE* = 0.060, *p* < .05). Further, while DP showed significant (p_{corr} < .05) increases in cortical oxygenation from control task 1 to control task 2 (mean difference = -0.115, CI [-0.219; -0.010], *SE* = 0.053, *p* < .05), as well as from control task 1 to the TSST (mean difference = -0.179, CI [-0.291; -0.067], *SE* = 0.057, *p* < .01), HC showed decreases between control task 1 and control task 2 (mean difference= 0.145, CI [0.031; 0.259], *SE* = 0.058, *p* < .05) and significant increases between control task 2 and the TSST (mean difference = -0.139, CI [-0.259; -0.031], *SE* = 0.053, *p* < .01).

Lastly, we observed significant lower-order main effects of ROI (O_2Hb: *F*(3.370, 471.745) = 10.995, *p* < .001, η_p^2 = .073, HHb: *F*(2.742, 375.640) = 3.443, *p* < .05, η_p^2 = .025) and condition (O_2Hb: *F*(2, 280) = 3.941, *p* < .05, η_p^2 = .027, HHb: *F*(2, 274) = 9.003, *p* < .001, η_p^2 = .062) indicating general increases in cortical oxygenation from the control tasks to the TSST.

Direct comparison of results with and without inclusion of age as a covariate:

Stress-related brain activation.

	analysis without inclusion of age		analysis with inclusion of age	
window 3	O_2Hb	HHb	O_2Hb	HHb
condition*group	*p* < .01	*p* = .424	*p* < .01	*p* = .348
ROI	*p* < .001	*p* < .05	*p* = .061	*p* = .248
condition	*p* < .001	*p* < .001	*p* < .05	*p* = .679
window 2				
condition*group	*p* = .155	*p* = .318	*p* = .353	*p* = .294
ROI	*p* < .001	*p* < .05	*p* < .001	*p* = .340
condition	*p* < .001	*p* < .001	*p* < .01	*p* < .01

Note. This table only compares the reported significant effects.

Anticipation-related brain activation.

	analysis without inclusion of age		analysis with inclusion of age	
window 1	O_2Hb	HHb	O_2Hb	HHb
condition*group	*p* < .01	*p* = .247	*p* < .05	*p* = .377
condition*ROI	*p* < .001	*p* = .075	*p* = .172	*p* = .508
ROI*group	*p* < .05	*p* = .241	*p* < .05	*p* = .429
ROI	*p* < .001	*p* < .05	*p* < .01	*p* = .052
condition	*p* < .05	*p* < .001	*p* = .154	*p* = .818

Note. This table only compares the reported significant effects.

Supplementary material S8: Details on ICD-10 diagnoses of DP

Table S8: Absolute and relative frequencies of diagnoses in DP

	DP study 1	DP study 2	All DP
F32.X	6 (27.3%)	48 (87.3%)	54 (70.1%)
F33.X	15 (68.2%)	6 (10.9%)	21 (27.3%)
F43.2	1 (4.5%)	1 (1.8%)	2 (2.6%)

Supplementary material S9: Details on the manipulation check

Subjective stress: Mahalanobis distances indicated 6 multivariate outliers (p < .001) and 1 missing data entry, resulting in a sample of 135 participants (n = 65 HC and n = 70 DP).

Fitting a rmANOVA for stress ratings as a function of condition and group, we observed a significant interaction of condition and group, F(3.999, 531.878) = 3.398, p < .01, η_p^2 = .025, as well as significant lower-order main effects of condition, F(3.999, 531.878) = 190.333, p < .001, η_p^2 = .589, and group, F(1, 133) = 77.921, p < .001, η_p^2 = .369.

The significant interaction indicated that while both groups exhibited significant increases in subjective stress following the stress induction, DP showed greater increases in response to the TSST than HC. Benjamini-Hochberg corrected pairwise comparisons between both groups indicated significant differences (p_{corr} < .05) between both groups during all conditions. Benjamini-Hochberg corrected pairwise comparisons of within each group indicated significant differences (p_{corr} < .05) between all consecutive conditions in each group except for between baseline and post rest 1 in DP and between 45 min and 60 min post TSST in HC (see figure S9A).

Positive affect: Mahalanobis distances indicated 1 multivariate outlier (p < .001), resulting in a sample of 141 participants (n = 65 HC and n = 76 DP).

Fitting the same rmANOVA for positive affect (PANAS PA), we only observed a significant main effect of group, F(1, 139) = 38.595, p < .001, η_p^2 = .217, indicating generally higher positive affect in HC compared to DP (see figure S9B).

Negative affect: Mahalanobis distances indicated 2 multivariate outliers (p < .001), resulting in a sample of 140 participants (n = 65 HC and n = 75 DP).

Fitting the rmANOVA for negative affect (PANAS NA), we observed a significant interaction of condition and group, F(1, 138) = 4.915, p < .05, η_p^2 = .028, as well as significant lower-order main effects of condition, F(1, 138) = 120.971, p < .001, η_p^2 = .467, and group, F(1, 138) = 85.269, p < .001, η_p^2 = .382. The significant interaction indicated that DP showed greater increases in negative affect than HC. Specifically, Benjamini-Hochberg corrected pairwise comparisons between both groups indicated significant differences (p_{corr} < .05) between both groups post rest1 and post TSST and Benjamini-Hochberg corrected pairwise comparisons of within each group indicated significant increases (p_{corr} < .05) between post rest1 and post TSST in each group (see figure S9C).

State rumination: Mahalanobis distances indicated 0 multivariate outliers ($p < .001$), resulting in a sample of 142 participants (n = 65 HC and n = 77 DP).

Fitting the same rmANOVA for state rumination, we again found an interaction of condition and group, $F(1, 140) = 6.125$, $p < .05$, $\eta_p^2 = .042$, as well as significant lower-order main effects of condition, $F(1, 140) = 46.249$, $p < .001$, $\eta_p^2 = .248$, and group, $F(1, 140) = 254.735$, $p < .001$, $\eta_p^2 = .645$. The significant interaction indicated that DP showed greater increases in state rumination than HC. Specifically, Benjamini-Hochberg corrected pairwise comparisons between both groups indicated significant differences ($p_{corr} < .05$) between both groups during rest1 and rest2 and Benjamini-Hochberg corrected pairwise comparisons of within each group indicated significant increases ($p_{corr} < .05$) between rest1 and rest2 in each group (see figure S9D).

Salivary cortisol: We further investigated salivary cortisol, we observed a significant main effect of condition, $F(1.887, 220.788) = 55.575$, $p < .001$, $\eta_p^2 = .322$, indicating TSST-related increases in salivary cortisol, but no significant main effect of group or interaction of group and condition (all p's > .140) (see figure S9E).

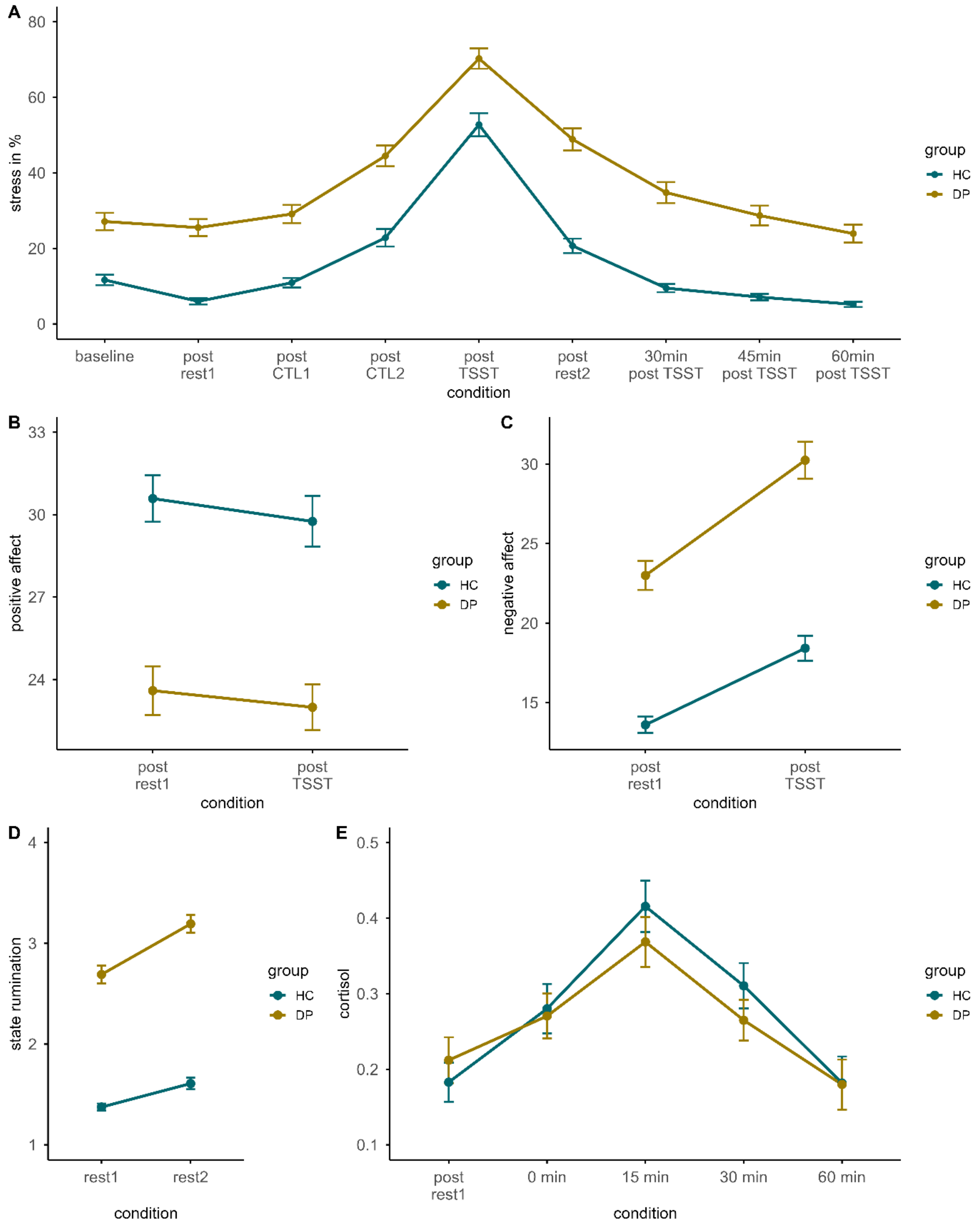

Figure S9. Line plot of stress (assessed using a Visual Analogue Scale ranging from 0 to 100%) (A), positive affect (PANAS PA) (B), negative affect (PANAS NA) (C), state rumination (assessed using the Stress-Reactive State Rumination questionnaire; Int-Veen et al., n.d.) (D) and salivary cortisol levels (E) dependent on condition (CTL1 = control task 1, i.e. reading numbers; CTL2 = control task 2, i.e. mental arithmetics without TSST panel; TSST = arithmetic task of the TSST, i.e. mental arithmetics with TSST panel) for two groups: HC (healthy controls) and DP (patients with depression). Lines depict the mean ratings for each group, error bars represent +/- 1 standard error of the mean (SE).

Supplementary material S10: Time series during the arithmetic task of the TSST

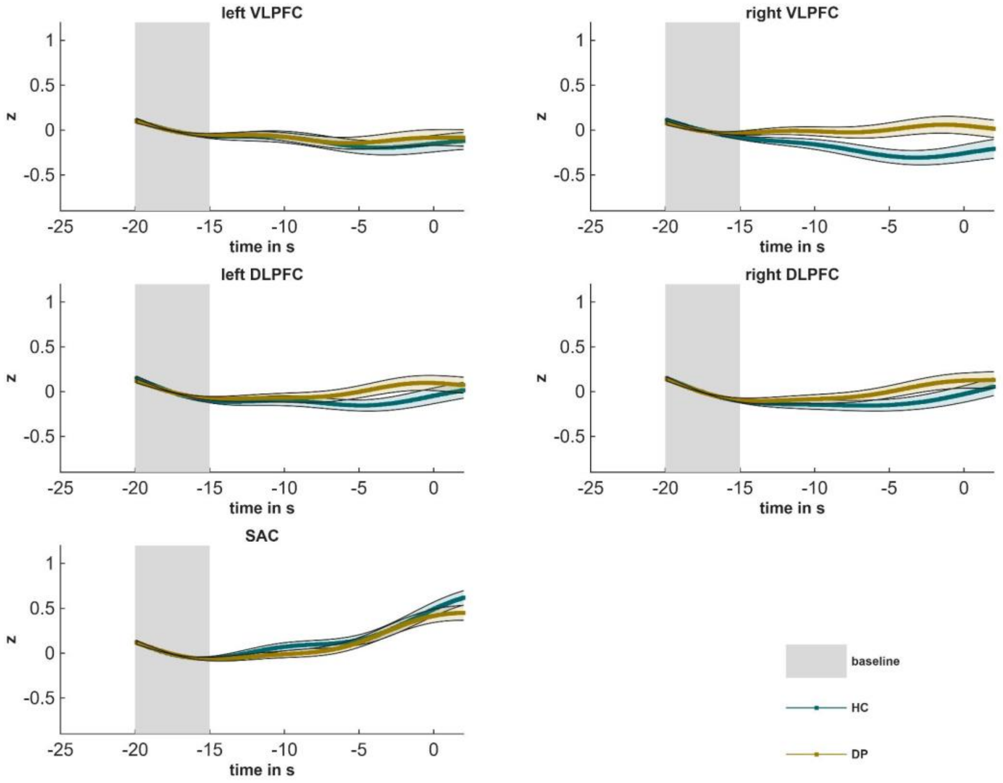

Figure S10.1. Illustration of the z-standardized hemodynamic responses during the arithmetic task of the TSST for two groups: HC (healthy controls) and DP (patients with depression) during the anticipation phase (window 1 figure 2) dependent on the Region of Interest (left and right IFG, left and right DLPFC and SAC). The dark shading marks the 5 s baseline. The investigated time window of the anticipation phase ranges from 15 s to 0 s prior to the trial. 0 s on the x-axis marks the beginning of the trial. Shadings around the hemodynamic curves reflect +/-1 standard error of the mean. IFG = Inferior Frontal Gyrus, DLPFC = Dorsolateral Prefrontal Cortex, SAC = Somatosensory Association Cortex.

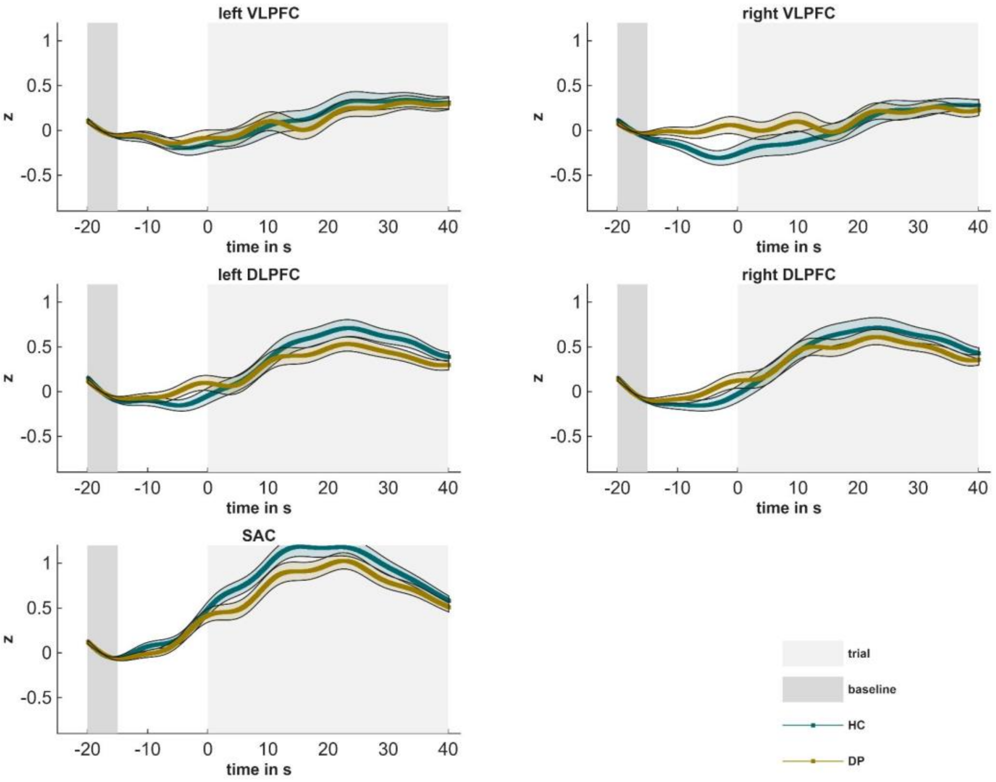

Figure S10.2. Illustration of the z-standardized hemodynamic responses during the arithmetic task of the TSST for two groups: HC (healthy controls) and DP (patients with depression) during the anticipation phase (window 2 figure 2) dependent on the Region of Interest (left and right IFG, left and right DLPFC and SAC). The light shading marks the 40 s trial (i.e. the exported time window 2) and the dark shading the 5 s baseline. 0 s on the x-axis marks the beginning of the trial. Shadings around the hemodynamic curves reflect +/-1 standard error of the mean. IFG = Inferior Frontal Gyrus, DLPFC = Dorsolateral Prefrontal Cortex, SAC = Somatosensory Association Cortex.

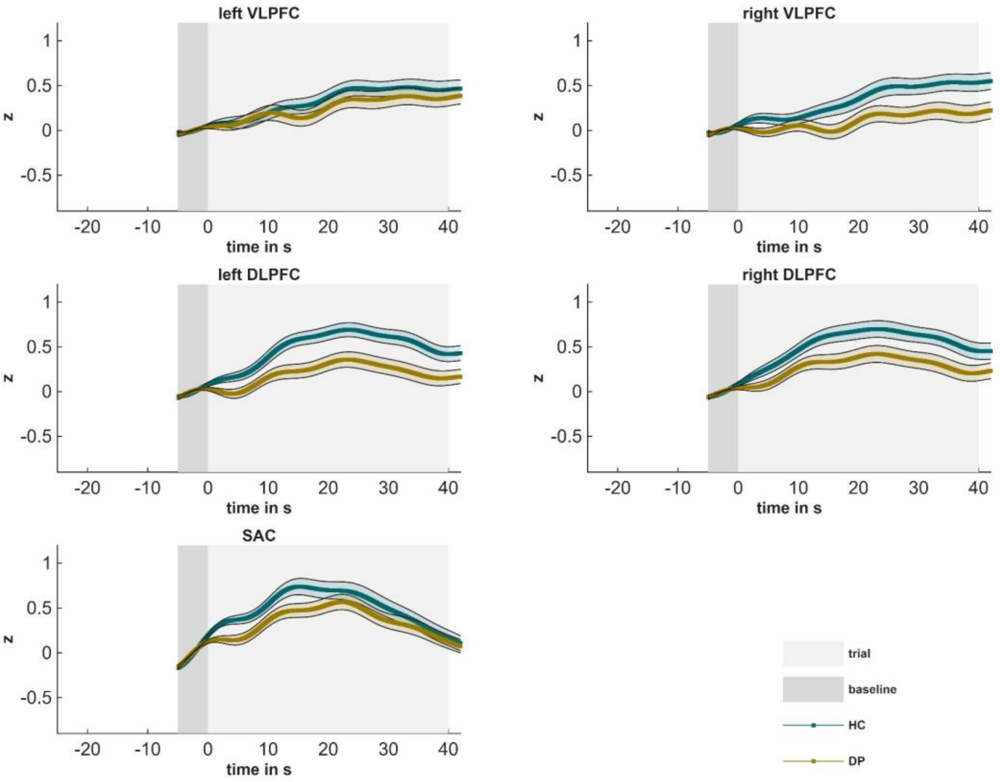

Figure S10.3. Illustration of the z-standardized hemodynamic responses during the arithmetic task of the TSST for two groups: HC (healthy controls) and DP (patients with depression) during the anticipation phase (window 3 figure 2) dependent on the Region of Interest (left and right IFG, left and right DLPFC and SAC). The light shading marks the 40 s trial (i.e. the exported time window 2) and the dark shading the 5 s baseline. 0 s on the x-axis marks the beginning of the trial. Shadings around the hemodynamic curves reflect +/-1 standard error of the mean. IFG = Inferior Frontal Gyrus, DLPFC = Dorsolateral Prefrontal Cortex, SAC = Somatosensory Association Cortex.

Supplementary material S11: Correlations of task-related neural activation (window 1, window 2 and 3) with subjective and physiological stress responses for HC and DP

window 1 (anticipation-related brain activation)

Table S11.1. Two-sided Pearson correlations investigating the association of window 1 neural activation and different physiological and behavioral variables for HC.

	left DLPFC	right IFG	right DLPFC	SAC	cortisol	heart rate	NA	SRSRQ	stress
left IFG	**.636*** **	**.486*** **	**.456*** **	**.315***	.204	-.186	.039	**.286***	.021
left DLPFC		**.403*** **	**.803*** **	**.616*** **	.059	-.233	-.017	.277*	.070
right IFG			**.509*** **	.199	-.021	-.300*	-.032	.159	-.073
right DLPFC				**.590** **	.050	-.280*	-.023	.268*	.005
SAC					-.022	-.128	-.002	.138	.053
cortisol						-.137	.049	.131	-.111
heart rate							.080	-.084	.071
NA								**.480*** **	**.575*** **
SRSRQ									**.417*** **

Note. Numbers represent correlation coefficients, asterisks represent uncorrected significance levels. * = $p < .05$, ** = $p < .01$, *** = $p < .001$, IFG = Inferior Frontal Gyrus, DLPFC = Dorsolateral Prefrontal Cortex, SAC = Somatosensory Association Cortex, heart rate = heart rate in beats per minute during the arithmetic task of the TSST, cortisol = salivary cortisol 15 min after the TSST, NA = negative affect scale of the PANAS 0 min after the TSST, SRSRQ = Stress-Reactive State Rumination Questionnaire after resting-state 2, Stress = subjective stress assessed using a Visual Analogue Scale 0 min after the TSST. The gray shading of the table cell indicates a significant correlation in window 1 for the total sample (see table 2 in the manuscript). Bold correlations remain significant at $p < .05$ when correcting row-wise for multiple comparisons using the Benjamini-Hochberg procedure.

Table S11.2. Two-sided Pearson correlations investigating the association of window 1 neural activation and different physiological and behavioral variables for DP.

	left DLPFC	right IFG	right DLPFC	SAC	cortisol	heart rate	NA	SRSRQ	stress
left IFG	**.680*** **	**.483*** **	**.509*** **	**.314** **	-.046	-.108	.103	.072	.125
left DLPFC		**.492*** **	**.791*** **	**.563*** **	-.187	-.084	.152	.182	.137
right IFG			**.514*** **	**.402*** **	-.110	-.107	.079	.162	.198
right DLPFC				**.574*** **	-.093	-.040	.048	.158	.095
SAC					-.031	.026	.074	.087	.144
cortisol						.267*	-.045	-.097	-.037
heart rate							.012	-.166	-.164
NA								**.631*** **	**.666*** **
SRSRQ									**.422*** **

Note. Numbers represent correlation coefficients, asterisks represent uncorrected significance levels. * = *p* < .05, ** = *p* < .01, *** = *p* < .001, IFG = Inferior Frontal Gyrus, DLPFC = Dorsolateral Prefrontal Cortex, SAC = Somatosensory Association Cortex, heart rate = heart rate in beats per minute during the arithmetic task of the TSST, cortisol = salivary cortisol 15 min after the TSST, NA = negative affect scale of the PANAS 0 min after the TSST, SRSRQ = Stress-Reactive State Rumination Questionnaire after resting-state 2, Stress = subjective stress assessed using a Visual Analogue Scale 0 min after the TSST. The gray shading of the table cell indicates a significant correlation in window 1 for the total sample (see table 2 in the manuscript). Bold correlations remain significant at *p* < .05 when correcting row-wise for multiple comparisons using the Benjamini-Hochberg procedure.

window 2 (task-related brain activation with baseline accounting for different baselines)

Table S11.3. Two-sided Pearson correlations investigating the association of window 2 neural activation and different physiological and behavioral variables for HC.

	left DLPFC	right IFG	right DLPFC	SAC	cortisol	heart rate	NA	SRSRQ	stress
left IFG	**.633*** **	**.649*** **	**.484*** **	**.416*** **	-.046	-.181	-.164	-.175	-.090
left DLPFC		**.400*** **	**.684*** **	**.521*** **	-.083	-.141	-.272*	-.055	-.060
right IFG			**.569*** **	.248*	-.057	-.121	-.165	-.054	-.093
right DLPFC				**.627*** **	-.118	-.101	-.266*	-.028	-.093
SAC					.025	-.130	-.111	-.110	-.001
cortisol						-.137	-.049	.131	-.111
heart rate							.080	-.084	.071
NA								**.480*** **	**.575*** **
SRSRQ									**.417*** **

Note. Numbers represent correlation coefficients, asterisks represent uncorrected significance levels. * = $p < .05$, ** = $p < .01$, *** = $p < .001$, IFG = Inferior Frontal Gyrus, DLPFC = Dorsolateral Prefrontal Cortex, SAC = Somatosensory Association Cortex, heart rate = heart rate in beats per minute during the arithmetic task of the TSST, cortisol = salivary cortisol 15 min after the TSST, NA = negative affect scale of the PANAS 0 min after the TSST, SRSRQ = Stress-Reactive State Rumination Questionnaire after resting-state 2, Stress = subjective stress assessed using a Visual Analogue Scale 0 min after the TSST. The gray shading of the table cell indicates a significant correlation in window 2 for the total sample (see table S5.1). Bold correlations remain significant at $p < .05$ when correcting row-wise for multiple comparisons using the Benjamini-Hochberg procedure.

Table S11.4. Two-sided Pearson correlations investigating the association of window 2 neural activation and different physiological and behavioral variables for DP.

	left DLPFC	right IFG	right DLPFC	SAC	cortisol	heart rate	NA	SRSRQ	stress
left IFG	**.588*** **	**.444*** **	**.521*** **	**.252***	**-.372** **	-.057	-.201	-.133	-.031
left DLPFC		**.529*** **	**.673*** **	**.459*** **	**-.295***	-.070	-.109	-.078	-.046
right IFG			**.607*** **	.197	**-.317***	**-.275***	-.014	.070	.162
right DLPFC				**.472*** **	-.179	-.054	-.072	.074	-.009
SAC					-.014	-.056	-.074	-.180	-.090
cortisol						.267*	-.045	-.097	-.037
heart rate							.012	-.166	-.164
NA								**.631*** **	**.666*** **
SRSRQ									**.422*** **

Note. Numbers represent correlation coefficients, asterisks represent uncorrected significance levels. * = *p* < .05, ** = *p* < .01, *** = *p* < .001, IFG = Inferior Frontal Gyrus, DLPFC = Dorsolateral Prefrontal Cortex, SAC = Somatosensory Association Cortex, heart rate = heart rate in beats per minute during the arithmetic task of the TSST, cortisol = salivary cortisol 15 min after the TSST, NA = negative affect scale of the PANAS 0 min after the TSST, SRSRQ = Stress-Reactive State Rumination Questionnaire after resting-state 2, Stress = subjective stress assessed using a Visual Analogue Scale 0 min after the TSST. The gray shading of the table cell indicates a significant correlation in window 2 for the total sample (see table S5.1). Bold correlations remain significant at *p* < .05 when correcting row-wise for multiple comparisons using the Benjamini-Hochberg procedure.

window 3 (task-related brain activation with standard baseline)

Table S11.5. Two-sided Pearson correlations investigating the association of window 3 neural activation and different physiological and behavioral variables for HC.

	left DLPFC	right IFG	right DLPFC	SAC	cortisol	heart rate	NA	SRSRQ	stress
left IFG	**.517*** **	**.445*** **	**.333** **	.182	-.151	.041	-.171	**-.426*** **	-.089
left DLPFC		.244	**.541*** **	**.512*** **	-.120	.211	-.273*	**-.377** **	-.195
right IFG			**.530** **	.112	-.010	.228	-.240	**-.305***	-.155
right DLPFC				**.535*** **	-.116	.189	**-.330** **	**-.299***	-.204
SAC					.037	.010	-.096	-.218	-.085
cortisol						-.137	.049	.131	-.111
heart rate							.080	-.084	.071
NA								**.480*** **	**.575*** **
SRSRQ									**.417*** **

Note. Numbers represent correlation coefficients, asterisks represent uncorrected significance levels. * = p < .05, ** = p < .01, *** = p < .001, IFG = Inferior Frontal Gyrus, DLPFC = Dorsolateral Prefrontal Cortex, SAC = Somatosensory Association Cortex, heart rate = heart rate in beats per minute during the arithmetic task of the TSST, cortisol = salivary cortisol 15 min after the TSST, NA = negative affect scale of the PANAS 0 min after the TSST, SRSRQ = Stress-Reactive State Rumination Questionnaire after resting-state 2, Stress = subjective stress assessed using a Visual Analogue Scale 0 min after the TSST. The gray shading of the table cell indicates a significant correlation in window 3 for the total sample (see table S5.2). Bold correlations remain significant at p < .05 when correcting row-wise for multiple comparisons using the Benjamini-Hochberg procedure.

Table S11.6. Two-sided Pearson correlations investigating the association of window 3 neural activation and different physiological and behavioral variables for DP.

	left DLPFC	right IFG	right DLPFC	SAC	cortisol	heart rate	NA	SRSRQ	stress
left IFG	**.491*** **	**.525*** **	**.360** **	.173	-.263*	.145	-.126	-.146	-.109
left DLPFC		**.573*** **	**.697*** **	**.627*** **	.022	.078	-.206	**-.260***	-.197
right IFG			**.534*** **	**.329** **	-.198	-.060	-.079	-.192	-.116
right DLPFC				**.479*** **	-.068	.036	-.020	-.086	-.090
SAC					.084	-.056	-.149	**-.293** **	-.264*
cortisol						.267*	-.045	-.097	-.037
heart rate							.012	-.166	-.164
NA								**.631*** **	**.666*** **
SRSRQ									**.422*** **

Note. Numbers represent correlation coefficients, asterisks represent uncorrected significance levels. * = $p < .05$, ** = $p < .01$, *** = $p < .001$, IFG = Inferior Frontal Gyrus, DLPFC = Dorsolateral Prefrontal Cortex, SAC = Somatosensory Association Cortex, heart rate = heart rate in beats per minute during the arithmetic task of the TSST, cortisol = salivary cortisol 15 min after the TSST, NA = negative affect scale of the PANAS 0 min after the TSST, SRSRQ = Stress-Reactive State Rumination Questionnaire after resting-state 2, Stress = subjective stress assessed using a Visual Analogue Scale 0 min after the TSST. The gray shading of the table cell indicates a significant correlation in window 3 for the total sample (see table S5.2). Bold correlations remain significant at $p < .05$ when correcting row-wise for multiple comparisons using the Benjamini-Hochberg procedure.

Supplementary material S12: Analysis using LSAS (social anxiety as a grouping variable)

		LSAS group		total
		low LSAS	high LSAS	
group	HC	49	15	64
	DP	11	65	76
total		60	80	140

Note that LSAS data was missing in two participants. We used a cutoff score of 30 to differentiate individuals with social anxiety disorder (SAD) from non-anxious individuals (Rytwinski et al., 2009).

Anticipation-related brain activation (window 1). Fitting a rmANOVA to investigate the neural activation in LSAS groups prior to the onset of the trial, there was a significant interaction of condition and ROI in O_2Hb, $F(6.023, 787.376) = 8.144$, $p < .001$, $\eta_p^2 = .056$ (HHb: $F(6.090, 822.111) = 1.875$, $p = .081$, $\eta_p^2 = .014$), as well as significant lower-order main effects of ROI for O_2Hb: $F(3.346, 461.787) = 9.833$, $p < .001$, $\eta_p^2 = .067$ (HHb: $F(2.674, 360.969) = 2.632$, $p = .056$, $\eta_p^2 = .019$) and condition (O_2Hb: $F(2, 276) = 4.043$, $p < .05$, $\eta_p^2 = .028$, HHb: $F(2, 270) = 9.478$, $p < .001$, $\eta_p^2 = .066$) indicating general increases in cortical oxygenation from the control tasks to the TSST.

There were no significant interaction or main effects of LSAS group (all *p*'s > .199).

Stress-related brain activation (window 2). Fitting a rmANOVA to investigate the neural activation in LSAS groups during the trial while correcting for brain activation prior to the trial, there was a significant interaction of condition and ROI (O_2Hb: $F(6.472, 893.116) = 15.051$, $p < .001$, $\eta_p^2 = .098$, HHb: $F(6.069, 825.432) = 8.959$, $p < .001$, $\eta_p^2 = .062$), as well as significant lower-order main effects of ROI (O_2Hb: $F(3.432, 473.658) = 61.197$, $p < .001$, $\eta_p^2 = .307$, HHb: $F(3.296, 448.288) = 3.373$, $p < .05$, $\eta_p^2 = .024$) and condition (O_2Hb: $F(2, 276) = 36.743$, $p < .001$, $\eta_p^2 = .210$, HHb: $F(2, 272) = 38.745$, $p < .001$, $\eta_p^2 = .222$) indicating general increases in cortical oxygenation from the control tasks to the TSST.

There were no significant interaction or main effects of LSAS group (all *p*'s > .071).

Stress-related brain activation (window 3). Fitting a rmANOVA to investigate the neural activation in LSAS groups during the trial with a standard baseline, there was a significant main effect of ROI (O_2Hb: $F(3.379, 466.279) = 11.295$, $p < .001$, $\eta_p^2 = .076$, HHb: $F(3.305, 449.505) = 3.122$, $p < .05$, $\eta_p^2 = .022$) and condition (O_2Hb: $F(2, 276) = 9.190$, $p < .001$, $\eta_p^2 = .062$, HHb: $F(2, 272) = 7.861$, $p < .001$, $\eta_p^2 = .055$) indicating general increases in cortical oxygenation from the control tasks to the TSST.

There were no significant interaction or main effects of LSAS group (all *p*'s > .054).

References of the Supplementary Material

First, M., Williams, J., Karg, R., & Spitzer, R. (2015). *Structured Clinical Interview for DSM-5 Disorders, Clinical Trials Version (SCID-5-CT)*. American Psychiatric Association.

Kligfield, P., Gettes, L. S., Bailey, J. J., Childers, R., Deal, B. J., Hancock, E. W., Van Herpen, G., Kors, J. A., Macfarlane, P., Mirvis, D. M., & others. (2007). Recommendations for the standardization and interpretation of the electrocardiogram: Part I: the electrocardiogram and its technology a scientific statement from the American Heart Association Electrocardiography and Arrhythmias Committee, Council on Clinical Cardiology; the American College of Cardiology Foundation; and the Heart Rhythm Society endorsed by the International Society for Computerized Electrocardiology. *Journal of the American College of Cardiology*, *49*(10), 1109–1127.

Laicher, H., Int-Veen, I., Woloszyn, L., Wiegand, A., Kroczek, A., Sippel, D., Leehr, E. J., Lawyer, G., Albasini, F., Frischholz, C., & others. (2023). In situ fNIRS measurements during cognitive behavioral emotion regulation training in rumination-focused therapy: A randomized-controlled trial. *NeuroImage: Clinical*, *40*, 103525.

Okamoto, M., Dan, H., Sakamoto, K., Takeo, K., Shimizu, K., Kohno, S., Oda, I., Isobe, S., Suzuki, T., Kohyama, K., & others. (2004). Three-dimensional probabilistic anatomical cranio-cerebral correlation via the international 10–20 system oriented for transcranial functional brain mapping. *Neuroimage*, *21*(1), 99–111.

Okamoto, M., & Dan, I. (2005). Automated cortical projection of head-surface locations for transcranial functional brain mapping. *Neuroimage*, *26*(1), 18–28.

Rosenbaum, D., Int-Veen, I., Laicher, H., Torka, F., Kroczek, A., Rubel, J., Lawyer, G., Bürger, Z., Bihlmaier, I., Storchak, H., & others. (2021). Insights from a laboratory and naturalistic investigation on stress, rumination and frontal brain functioning in MDD: An fNIRS study. *Neurobiology of Stress*, 100344.

Rosenbaum, D., Int-Veen, I., Laicher, H., Woloszyn, L., Wiegand, A., Ladegast, S., Eßer, U., Kroczek, A., Sippel, D., Menkor, S., & others. (2024). Neural correlates of stress-reactive rumination in depression–the role of childhood trauma and social anxiety. *Neurobiology of Stress*, 100640.

Rytwinski, N. K., Fresco, D. M., Heimberg, R. G., Coles, M. E., Liebowitz, M. R., Cissell, S., Stein, M. B., & Hofmann, S. G. (2009). Screening for social anxiety disorder with the self-report version of the Liebowitz Social Anxiety Scale. *Depression and Anxiety*, *26*(1), 34–38. https://doi.org/10.1002/da.20503.

Singh, A. K., Okamoto, M., Dan, H., Jurcak, V., & Dan, I. (2005). Spatial registration of multichannel multi-subject fNIRS data to MNI space without MRI. *Neuroimage*, *27*(4), 842–851.
